# Glutaminase as a metabolic target of choice to counter acquired resistance to Palbociclib by colorectal cancer cells

**DOI:** 10.1038/s41388-025-03495-w

**Published:** 2025-07-22

**Authors:** Míriam Tarrado-Castellarnau, Carles Foguet, Josep Tarragó-Celada, Marc Palobart, Claudia Hernández-Carro, Jordi Perarnau, Erika Zodda, Ibrahim H. Polat, Silvia Marin, Alejandro Suarez-Bonnet, Juan José Lozano, Mariia Yuneva, Timothy M. Thomson, Marta Cascante

**Affiliations:** 1 Department of Biochemistry and Molecular Biomedicine and Institute of Biomedicine (IBUB), Universitat de Barcelona, Barcelona, Spain; 2 CIBER of Hepatic and Digestive Diseases (CIBEREHD), Institute of Health Carlos III (ISCIII), Madrid, Spain; 3 British Heart Foundation Cardiovascular Epidemiology Unit and Victor Phillip Dahdaleh Heart and Lung Research Institute, University of Cambridge, Cambridge, UK; 4 Oncogenes and Tumour Metabolism Laboratory, The Francis Crick Institute, London, UK; 5 Institute of Molecular Biology of Barcelona (IBMB-CSIC), Barcelona, Spain; 6 Experimental Histopathology, The Francis Crick Institute, London, UK; 7 Department of Pathobiology and Population Sciences, The Royal Veterinary College, Hatfield, UK; 8 Bioinformatics Platform, Centro de Investigación Biomédica en Red de Enfermedades Hepáticas y Digestivas (CIBEREHD), Barcelona, Spain; 9 Instituto de Investigaciones de la Altura, Universidad Peruana Cayetano Heredia, Lima, Perú; 10 Instituto de Investigaciones Científicas y Servicio de Alta Tecnología (INDICASAT AIP), Panamá City, Panamá

**Keywords:** Cancer therapeutic resistance, Target validation, Cancer metabolism

## Abstract

Several mechanisms of resistance of cancer cells to cyclin-dependent kinase inhibitors (CDKi) have been identified, including the upregulation of metabolic regulators such as glutaminase. However, whether such resistance mechanisms represent optimal targets has not been determined. Here, we have systematically analyzed metabolic reprogramming in colorectal cancer cells exposed to Palbociclib, a CDKi selectively targeting CDK4/6, or Telaglenastat, a selective glutaminase inhibitor. Through multiple approaches, we show that Palbociclib and Telaglenastat elicit complementary metabolic responses and are thus uniquely suited to counter the metabolic reprogramming induced by the reciprocal drug. As such, while Palbociclib induced reduced tumor growth in vivo, and Telaglenastat did not show a significant effect, the drug combination displayed a strong synergistic effect on tumor growth. Likewise, initial responses to Palbociclib were followed by signs of adaptation and resistance, which were prevented by combining Palbociclib with Telaglenastat. In conclusion, combination with Telaglenastat optimally forestalls acquired resistance to Palbociclib in cancer cells.

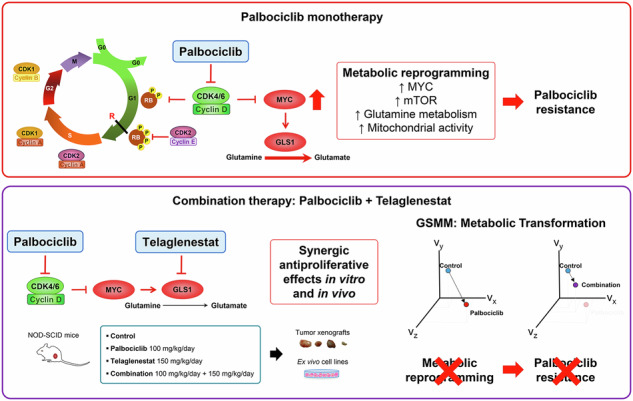

## Introduction

Cell cycle control is frequently dysregulated in cancer cells leading to unscheduled proliferation [1]. Cyclin-dependent kinases CDK4 and CDK6 (CDK4/6) are effective targets in cancer therapy since their overexpression and dysregulation are implicated in a wide range of human cancers [2, 3]. Three selective pharmacologic CDK4/6 inhibitors have received approval from the US Food and Drug Administration (FDA) and the European Medicines Agency (EMA) for the treatment of estrogen receptor (ER)-positive, human epidermal growth factor receptor 2 (HER2)-negative locally advanced or metastatic breast cancer [4]. Among these, Palbociclib (PD0332991) was the first-in-class drug to show promising results in phase III studies [5–7]. More recently, Palbociclib and other CDK4/6 inhibitors (CDK4/6i), in a variety of combinations, have shown efficacy in other cancer types, including colorectal carcinoma [8–24], particularly in tumors bearing genetic or epigenetic alterations underlying an upregulated G1-S cell cycle transition [22, 24]. In addition to a direct effect on cancer cell survival, CDK4/6i have been shown to promote anti-tumor immune responses [23, 25]. However, as single agents, CDK4/6i have shown limited efficacy outside of ER+ breast cancers [26, 27] and, like with most targeted therapies, tumor cells eventually acquire resistance to CDK4/6 inhibition [28–31]. Experimentally tested mechanisms of CDK4/6i resistance include gain-of-function changes impacting cell-cycle transitions [32–35], MYC dependency [29, 32, 36, 37], metabolic reprogramming [36, 38, 39], or other signaling and regulatory pathway adaptations [29, 40–43].

Our previous work characterized the metabolic reprogramming associated with CDK4/6 pharmacological inhibition or genetic depletion, uncovering the upregulation of MYC, glutaminolysis, and mTOR signaling as major adaptations of colorectal cancer cells to restore their fitness in response to such perturbations [36]. Our results demonstrated that these adaptations render cancer cells that survive CDK4/6 inhibition highly sensitive to MYC, glutaminase (GLS1), or mTOR inhibitors and hypoxic conditions. These observations warranted a deeper exploration of the mechanisms explaining the observed synergism between CDK4/6 and glutaminase inhibition.

Herein, we have approached this by analyzing transcriptomic and metabolomic responses elicited in colon cancer cells that persist after exposure either to the CDK4/6i Palbociclib or the glutaminase inhibitor Telaglenastat (CB-839), or their combination, in vitro and in vivo. The underlying hypothesis is that the observed responses may reflect resistance mechanisms that at least partly explain the emergence of persister cells. Cross-analysis of such responses revealed that upregulation of glutaminolysis is the most relevant metabolic response to Palbociclib treatment. Conversely, Telaglenastat elicits a prominent downregulation of the metabolic flux through oxidative phosphorylation (OXPHOS), glycolysis, and pentose phosphate pathway (PPP). Therefore, these two drugs elicit complementary responses in persister cells. These adaptive responses represent drug-specific emerging vulnerabilities, as demonstrated by the observation that Palbociclib-resistant cells become sensitive to Telaglenastat. Remarkably, we have found that residual cancer cells from tumors treated in vivo with the combined therapy remain sensitive to the dual treatment ex vivo, long after chemotherapeutic exposure, and exhibit a downregulated metabolism. Together, our results validate the combination of Palbociclib and Telaglenastat as an effective strategy to prevent pharmacologic resistance and potentiate the antiproliferative effects of single-agent treatments. A Catalan translation of the abstract and the graphical summary of this article can be found at 10.5281/zenodo.15876814.

## Methods

### Compounds and reagents

Palbociclib (PD0332991) and Telaglenastat (CB-839) were purchased from Selleckchem (Houston, TX, USA). The glutaminase inhibitor bis-2-(5-phenylacetamido-1,2,4-thiadiazol-2-yl)ethyl sulfide (BPTES) was kindly provided by Dr. Mariia Yuneva (The Francis Crick Institute, London, UK). The glutamine analog 6-Diazo-5-oxo-L-nor-Leucine (DON), the GAC inhibitor Compound 968, N-acetyl cysteine (NAC), and dimethyl α-ketoglutarate (DM-αKG) were obtained from Merck (Darmstadt, Deuchland). The KGA inhibitor Glutaminase-IN-1 was purchased from MedChem Express (Monmouth Junction, NJ, USA). The inhibitor of type I PRMTs GSK3368715 was purchased from Cayman Chemical (Ann Arbor, MI, USA). Stock solutions of 10 mM were prepared with water or dimethyl sulfoxide according to the manufacturer’s instructions. Antibiotics (10,000 U/ml penicillin, 10 mg/ml streptomycin), PBS, and Trypsin EDTA solution C (0.05% trypsin—0.02% EDTA) were obtained from Biological Industries (Kibbutz Beit Haemet, Israel), and fetal bovine serum (FBS) from Invitrogen (Carlsbad, CA, USA).

### Cell culture

The human colorectal carcinoma cell lines HCT116, SW403, HT29, and SW620, human breast adenocarcinoma cell lines MCF-7, SK-BR-3, and MDA-MB-231, and the human embryonic kidney 293T (HEK293T) cells were obtained through the American Type Culture Collection (ATCC, Manassas, VA, USA). The HCT116 derivative HCT116^Luciferase-GFP^ cells were obtained through lentiviral transfection and transduction. Twelve HCT116 derivative cell lines were obtained from tumors from mice that had been treated daily with vehicle, Palbociclib, Telaglenastat, or the combination of Palbociclib and Telaglenastat for 23 days (three independent cell lines per condition). All cell lines were grown in the absence of chemotherapeutics. Palbociclib long-term resistant cell line was established by culturing HCT116 cells in gradient concentrations of Palbociclib for 4 months. HCT116 and SK-BR-3 cells and all the derivative cell lines were cultured in Dulbecco’s modified Eagle medium (Gibco, Thermo Fisher Scientific Inc., Waltham, MA, USA)/Nutrient mixture HAM F12 (Biological Industries) (DMEM/F12, 1:1 mixture) with 2.5 mM L-glutamine and 12.5 mM D-glucose. SW403, HT29, SW620, MDA-MB-231, and HEK293T cells were cultured in DMEM culture medium. MCF-7 cells were cultured in MEM culture medium without phenol red (Gibco) and supplemented with 10 mM D-glucose, 2 mM L-glutamine, 1 mM pyruvate (Biological Industries), 0.01 mg/ml insulin and 1% non-essential amino acids (Biological Industries). Media were supplemented with 10% FBS, penicillin (50 U/ml), and streptomycin (50 µg/ml). Cells were incubated at 37 °C in a humidified atmosphere with 5% CO_2_. All cell lines were regularly tested for mycoplasma contamination and cultivated for less than 3 months after receipt, generation, or thawing.

### Lentiviral transfection and transduction

HEK293T cells were transiently transfected using Fugene reagent (Roche, Indianapolis, IN, USA) with pCMV-GFP/Luc plasmid for the constitutive co-expression of the firefly luciferase gene and GFP. Viral supernatants were harvested 48 h after transfection, filtered through 0.45 μm and 0.22 μm methylcellulose filters (Merck Millipore, Billerica, MA, USA), and used to transduce HCT116 recipient cells. 5 × 10^5^ HCT116 cells were cultured in the presence of the viral supernatant and 8 μg/ml polybrene for 24 h. GFP-positive transduced cells were selected by fluorescence-activated cell sorting (MoFlo, Beckman Coulter). Luminescence levels yielded by cell lysates were measured using the Luciferase Assay System kit (Promega, Madison, WI, USA) in a Mithras LB 940 plate reader (Berthold Technologies, Bad Wildbad, Germany).

### Cell proliferation assays

Having optimized the seeding density, 2 × 10^3^ cells per well were seeded in 96-well plates, and media were replaced at 24 h with fresh media containing different concentrations of each drug, drug combination, or vehicle. Plates were placed in an IncuCyte® S3 (Sartorius, Göttingen, Germany) live-cell analysis system or in an Olympus CM30 Incubation Monitoring System (Evident Corporation, Tokyo, Japan) to monitor cell confluence. At 96 h, cells were washed with PBS and lysed with 0.01% SDS, and plates were stored frozen at −20 °C overnight. Then, plates were thawed at 37 °C and incubated in darkness with 4 µg/ml Hoechst (HO33342; 2’-[4-ethoxyphenyl]-5-[4-methyl-1-piperazinyl]-2,5′-bi-1H-benzimidazole trihydrochloride trihydrate, Sigma-Aldrich, St Louis, MO, USA) DNA stain in solution buffer (1 M NaCl, 1 mM EDTA, 10 mM Tris-HCl pH 7.4) on a shaker at 37 °C for 1 h. Finally, fluorescence was measured in a FLUOstar OPTIMA microplate reader (BMG LABTECH GmbH, Ortenberg, Germany) at 355 nm excitation and 460 nm emission. Cell proliferation was represented as a percentage relative to untreated control cells. Concentrations that caused 50% inhibition of cell proliferation (IC_50_) were calculated using GraphPad Prism 7 software (GraphPad Software, San Diego, CA, USA). Cell size and volume values were obtained using a Scepter^TM^ Handheld Automated Cell Counter (EMD Millipore Corporation, Billerica, MA), which employs the Coulter principle of impedance-based particle detection.

### Dose-response assays and combination index determination

Drug synergies were evaluated with the CompuSyn software (ComboSyn, Inc., Paramus, NJ, USA). The effectiveness of the combined drug treatments was quantified by determining the combination index (CI), where CI < 1, CI = 1, and CI > 1 indicate synergism, additivity, and antagonism, respectively [44].

### Colony formation assays

Single-cell suspensions of 1 × 10^3^ cells per well were seeded in 6-well plates and treated after 24 h with either vehicle, single drugs, or their combination. Colonies were allowed to grow for 10 days and then fixed and stained with 0.5% (w/v) Crystal Violet (Sigma-Aldrich) in 20% methanol. Image acquisition with ImageJ software (public domain National Institutes of Health, USA, http://rsbweb.nih.gov/ij/) was used to score the number of colonies.

### Spheroid assays

A total of 1 × 10^4^ cells were seeded in 24-well ultra-low attachment culture plates (Corning, NY, USA) in the presence of the specified inhibitor(s) in serum-free media supplemented with 20 ng/ml EGF (Gibco), 20 ng/ml bFGF (Gibco), 10 µg/ml heparin (Sigma-Aldrich), 1:50 (v/v) B-27 supplement 50x (Gibco), 5 µg/ml human insulin (Sigma-Aldrich) and 0.5 µg/ml hydrocortisone (Sigma-Aldrich). At the end of the experiment, spheroids were incubated with 0.5 mg/ml 3-(4,5-dimethylthiazol-2-yl)-2,5-diphenyltetrazolium bromide (MTT, Sigma-Aldrich) for 2–3 h until fully stained. Plates were scanned, spheroids were scored, and spheroid area and volume were quantified by image acquisition with ImageJ software.

### Cell cycle analysis

Cells were collected by centrifugation after trypsinization, resuspended in PBS, and fixed dropwise with 70% (v/v) cold ethanol for at least 2 h. Then, cells were centrifuged, washed twice with PBS, resuspended in PBS containing 0.2 mg/ml DNAse free RNAse A (Roche, Basel, Switzerland), and incubated for 1 h at 37 °C. Prior to analysis, 50 µg/ml propidium iodide (PI) was added. Samples were analyzed using a Gallios multi-color flow cytometer instrument (Beckman Coulter, Brea, CA, USA) set up with the 3 lasers 10 colors standard configuration. Excitation was done with a blue (488 nm) laser. Forward scatter (FS), side scatter (SS), red (620/30 nm) fluorescence emitted by PI were collected. Red fluorescence from PI was projected on a 1024-channel monoparametric histogram. Aggregates were excluded by gating single cells by their area vs. peak fluorescence signal. DNA analysis (ploidy analysis) from 10^4^ cells on single fluorescence histograms was done using the MultiCycle software (Phoenix Flow Systems, San Diego, CA, USA).

### Western blot analysis

Cells were washed twice with ice-cold PBS and incubated for 30 min on ice with RIPA buffer containing 50 mM Tris (pH 8.0), 150 mM sodium chloride, 1% Triton X-100, 0.5% sodium deoxycholate, 0.1% sodium dodecyl sulfate (SDS), 1% protease inhibitor cocktail set III and 1% phosphatase inhibitor cocktail set IV (EMD Millipore Corporation). Cells were scraped, sonicated, and centrifuged at 12,000 × *g* for 20 min at 4 °C. Supernatants were recovered, and the protein content was quantified with the BCA kit (Pierce Biotechnology).

Equal amounts of protein per sample were size-separated by electrophoresis on SDS-polyacrylamide gels and electroblotted onto 0.45 µm pore size Immobilon^®^-P polyvinylidene fluoride (PVDF) transfer membranes (Merck Millipore Ltd., Cork, IRL). After 1 h of blocking at room temperature with 5% skim milk in PBS 0.1% Tween, blots were incubated with specific primary antibodies overnight at 4 °C. Then, membranes were treated with the appropriate horseradish peroxidase (HRP)-labeled secondary antibody for 1 h at room temperature. All blots were treated with Immobilon^®^ Western Chemiluminescent HRP Substrate (EMD Millipore Corporation, Burlington, MA, USA) and developed after exposure to an autoradiography film (VWR International, Radnor, PA, USA). The primary antibodies used were Phospho-Akt (Ser473) (#9271), mTOR (#2972), and Phospho-mTOR (Ser2448) (#5536) from Cell Signaling (Beverly, MA, USA); GLS1 (ab93434), GLDH (ab166618) and MYC (ab32072) from Abcam (Cambridge, UK); GAC (19958-1-AP) and KGA (20170-1-AP) from Proteintech (Chicago, IL, USA); HIF1α (sc-13515) from Santa Cruz Biotechnology; and β-actin (#69100) form MP Biomedicals (Santa Ana, CA, USA). The secondary antibodies used were anti-mouse (ab6728) and anti-rabbit (ab6721) from Abcam.

### Measurement of extracellular metabolites

Media samples were collected at the beginning and the end of the incubation and frozen until analyzed. At the same time points, cell number was determined for normalization purposes. All the biochemical assays were carried out under exponential growth conditions. Glucose, lactate, glutamate, and glutamine concentrations from cell culture media were determined using a COBAS Mira Plus spectrophotometer (HORIBA ABX Diagnostics, Kyoto, Japan) to monitor the production of NAD(P)H in specific reactions for each metabolite at 340 nm wavelength. Glucose concentration was measured using hexokinase and glucose-6-phosphate dehydrogenase coupled enzymatic reactions (ABX Pentra Glucose HK CP, HORIBA ABX, Montpellier, France). Lactate concentration was determined at 37 °C with 2.35 mg/ml NAD^+^ (Roche) in 0.15 M hydrazine 10.9 mM EDTA pH 9 buffer and 80 U/ml lactate dehydrogenase (Roche) in 51 mM ammonium sulfate pH 6.5 buffer. Glutamate concentration was assessed at 37 °C with 2.08 mM ADP, 3.35 mM NAD^+^, and 16 U/ml of glutamate dehydrogenase (Roche) in 0.28 M glycine/0.35 M hydrazine/1.12 mM EDTA pH 9 buffer. Glutamine concentration was calculated through its conversion to glutamate at 37 °C for 30 min with 100 mU/ml glutaminase in 0.1 M acetate pH 5 buffer and subsequently quantifying glutamate concentration as described above.

### Estimation of metabolite consumption and production rates

Net fluxes per cell of uptake and release of different metabolites (*J*_*met*_) were estimated from the experimentally measured variations of metabolite concentration in medium and cell number for 24 h. The estimation was performed by assuming exponential growth and constant uptake or release per cell, which corresponds to a simple model of cell growth and metabolite consumption/production:

1 $$\frac{d{N}_{t}}{{dt}}\,=\,{N}_{t}\times \mu$$

2 $$\,\frac{d{M}_{t}}{{dt}}={N}_{t}\times {J}_{{met}}$$

Where *N* is cell number, *M* is the quantity of metabolite, and *µ* growth rate.

### Metabolite extraction and determination by HPLC-MS analysis

Cells were seeded in 6-well plates, incubated for 96 h with the treatments, and metabolites were extracted immediately after the endpoint. Cells were first washed twice with 1 ml ice-cold PBS, added 150 μl of methanol and 150 μl of water (containing 3 nmol [U-^13^C,^15^N] valine per sample as internal standard). Then, cells were scraped and transferred to a chilled 2 ml Eppendorf tube containing 300 μl of chloroform. After vortexing (3 pulses of 5 s each), and sonicating (3 times for 8 min), the tubes were incubated for 1 h at 4 °C, and finally centrifuged at 21,000 × *g*, 4 °C for 10 min. Supernatants containing the polar metabolite extract phase were collected and submitted for HPLC-MS analysis.

Polar metabolite HPLC-MS analysis was performed on a Q Exactive Plus mass spectrometer (Thermo Fisher Scientific Inc.). The chromatographic separation was performed on a SeQuant® Zic®-pHILIC (Merck Millipore) column (5 μm particle size, polymeric, 150 × 4.6 mm). The injection volume was 10 μl, and chromatographic separation was performed with a 300 μl/min flow rate, over a total time of 25 min. Mobile phases were ammonium carbonate 20 mM in water (A) and acetonitrile (B). The elution gradient was a decreasing percentage of B from 80 to 4% during 17 min, holding at 5% of B for 3 min, and finally re-equilibrating the column at 80% of B for 4 min. The mass resolving power was set to 70,000 for both analyses, and both negative and positive ion polarities were used for maximal metabolite coverage, and it was run in SCAN mode.

LC-MS data were analyzed using the Xcalibur software (Thermo Fisher Scientific Inc.) for validating metabolites and retention times, TraceFinder 4.1 (Thermo Fisher Scientific Inc.) for automatic detection and quantification of peaks. For each metabolite, the area under the curve was normalized to the area under the curve of the internal standard [U-^13^C,^15^N] valine and by protein.

### Determination of intracellular reactive oxygen species (ROS) levels

Cells were grown in dark, clear bottom 96-well microplates and treated for 96 h with the desired conditions. At the end of the treatments, cells were washed once with warm PBS, and incubated with 20 µM 2′-7′-dichlorodihydrofluorescein diacetate (H_2_DCFDA, Invitrogen) in PBS supplemented with 5.5 mM glucose. After 45 min at 37 °C and 5% CO_2_, the solution was replaced with PBS supplemented with 5.5 mM glucose for another 2 h at 37 °C and 5% CO_2_. Plates were measured in a FLUOstar OPTIMA microplate reader (BMG LABTECH GmbH, Ortenberg, Germany) at 485 nm excitation and 535 nm emission in end-point mode. Results were normalized by cell number.

### Total glutathione quantification

Fresh cells were lysed with 5% 5-sulfosalicylic acid solution, vortexed, and disrupted by two freezing/thawing cycles in liquid nitrogen and a 37 °C water bath. Cell extracts were incubated at 4 °C for 10 min and centrifuged at 10,000 × *g* for 10 min. The reaction was initiated by mixing 150 µl of working solution (15 U/ml glutathione reductase and 40 µg/ml 5,5′-Dithiobis(2-nitrobenzoic acid) in 100 mM K_2_HPO_4_/KH_2_PO_4_ 1 mM EDTA pH 7.0 buffer) with 10 µl of cell extract (diluted 1:5 or 1:10) or oxidized glutathione standards (from 0 to 12.5 µM). Then, 50 µl of 0.16 mg/ml NADPH solution were added to the samples, and the increase in absorbance with time was measured at 340 nm wavelength. Total glutathione concentration was normalized by protein content.

### Mouse xenografts and in vivo drug studies

Animal experiments were carried out at the Parc Científic de Barcelona—Parc de Recerca Biomèdica de Barcelona (PCB-PRBB) Animal Facility Alliance, in compliance with current regulations (RD 53/2013; Directive 2010/63/UE; Decree 214/1997/GC, Order ECC/566/2015), to ensure that animal experiments are conducted in a humane and ethical manner. The protocol for this study was approved by the PCB’s Animal Experimentation Ethics Committee and the Animal Experimentation Commission of the Generalitat de Catalunya.

Palbociclib isethionate (S1579, SelleckChem) was resuspended at 50 mg/ml in 50 mM sodium L-lactate (Sigma-Aldrich) pH 4 buffer. Telaglenastat (S7655, SelleckChem) was dissolved at 50 mg/ml in 10 mM sodium citrate (Sigma-Aldrich) pH 2 buffer with 25% w/v (2-hydroxypropyl)-β-cyclodextrin (HP-β-CD, Sigma-Aldrich). Treatments were aliquoted into single and combined daily doses and frozen at −80 °C. HCT116^Luciferase-GFP^ cells, after a test for mycoplasma contamination, were resuspended in sterile PBS and injected (10^6^ cells in 100 µl) subcutaneously on the right flank of anesthetized 5-week-old male non-obese diabetic (NOD) severe combined immunodeficiency disease (SCID) mice (strain: NOD.CB17/AlhnRj-Prkdcscid; type: Mutant congenic mouse; Janvier Labs, Le Genest-Saint-Isle, France). Sample size (*n* = 14 mice/group) was based on prior studies in similar models to ensure reproducibility and robustness of results. Tumor growth was monitored three times a week by non-invasive bioluminescence on an IVIS^®^ Spectrum In Vivo Imaging System (PerkinElmer, Waltham, MA, USA). Images and radiance were acquired 10 min after intraperitoneal injection of 150 µl of 100 mg/kg D-luciferin (BioVision Inc., Waltham, MA, USA) in sterile PBS. Eight days after tumor cell implantation, mice were randomly distributed in 4 treatment groups of fourteen animals each and administered via oral gavage with daily doses of vehicle, 100 mg/kg/day Palbociclib, 150 mg/kg/day Telaglenastat, or 100 mg/kg/day Palbociclib + 150 mg/kg/day Telaglenastat for 23 consecutive days. Mice were weighed every 3 days to readjust the treatment volume for precise drug dosing. Average radiance (photons (p)/s/cm^2^/steradian (sr)) was calculated for each mouse using a circular region of interest with the mouse in a prone position and normalized to the value obtained at the same area before starting treatment administrations (day 8) using the IVIS® Spectrum Living Image® 4.3.1 software (PerkinElmer). Tumor growth was also monitored thrice a week from day 15 by measuring tumor volume with a Vernier caliper using the modified ellipsoid volume formula: Tumor volume (mm^3^) = (length × width^2^) × π/6. Tumor growth inhibition (TGI) rate was calculated as TGI = (1 − (V_(T,t)_/V_(T,0)_)/(V_(C,t)_/V_(C,0)_)) × 100 [45], where time 0 is the first measurement of tumor volume on day 15. Researchers administering treatments and recording measurements (weight, tumor size, and radiance) were blinded to the expected results. At the end of the treatment, mice were euthanized, and residual tumors were collected for further analysis. Animals were allowed to form tumors up to 1.5 cm in diameter, at which point they were euthanized.

### Cell line generation ex vivo

Three tumors for each treatment group were excised, placed in Petri dishes with DMEM/F12 medium, cut into fragments with a sterile scalpel, and subjected to digestion with 1 mg/ml collagenase type IV (Sigma-Aldrich) for 30 min at 37 °C under aseptic conditions. Then, tissue was centrifuged and digested with Trypsin EDTA solution C to generate a single cell suspension, filtered through a 40 µm cell strainer, and plated in DMEM/F12 with 2.5 mM L-glutamine, 12.5 mM D-glucose, 10% FBS, penicillin (50 U/ml), and streptomycin (50 µg/ml), obtaining twelve independent cell lines (three for each treatment condition).

### Immunohistochemistry (IHC) staining of mouse xenograft tissues

Mouse xenografts were dissected immediately after surgical resection. Three tumors for each treatment condition were fixed in 4% paraformaldehyde (PFA) and paraffin-embedded in blocks for histology assessment. Serial 4-µm microtome sections were obtained from the PFA-fixed, paraffin-embedded tumor tissues. Immunohistochemistry staining was performed by the Francis Crick Institute (UK) Experimental Histopathology Laboratory on the Discovery Ultra Ventana platform (Roche). Antibody dilutions of 1:750 for CD31 (ab182981, Abcam) and of 1:1000 for KI67 (ab15580, Abcam) were used, with antigen retrieval using Cell Conditioning 1 (CC1, Roche) for 48 min and primary antibody incubation for 60 min. All slides were counterstained with Harris hematoxylin nuclear stains (Leica Biosystems, Wetzlar, Germany), dehydrated, cleared, and mounted in a Tissue-Tek Prisma® automated slide stainer (Sakura Finetek, Torrance, CA, USA). Slides were imaged on the Zeiss Axio Scan.Z1 slide scanner (Carl Zeiss Microscopy GmbH, Jena, Germany), and the percentage of KI67 and CD31 positive cells was evaluated using QuPath version 0.4.2 [46]. A board-certified pathologist (A.S-B.) assessed the mitotic index by counting mitoses in 10 high-power fields (HPF, 400×) on hematoxylin and eosin (H&E)-stained tumor sections.

### RNA sequencing

Total RNA was isolated using the RNeasy mini kit (Qiagen, Hilden, Germany) following the manufacturer’s instructions and including a DNase (Qiagen) digestion step. The size, concentration, and integrity of total RNA were determined by electrophoresis in a 4200 TapeStation system (Agilent, Santa Clara, CA, USA). Whole transcriptome sequencing (RNA-Seq) was performed at the Genomics Unit of CNIC (National Centre for Cardiovascular Research, Madrid, Spain). Barcoded RNA-Seq libraries were prepared with 200 ng of total RNA with RIN > 8 using the NEBNext® Ultra™ RNA Library Prep Kit for Illumina (New England Biolabs, Ipswich, MA, USA). Poly A + RNA was purified using poly-T oligo-attached magnetic beads followed by fragmentation and first and second cDNA strand synthesis. The second strand was synthesized with uracil instead of thymine. Then, cDNA 3′-ends were adenylated, adapters were ligated, uracils were excised, and the libraries were amplified by PCR. The size of the libraries was determined using a 2100 Bioanalyzer DNA 1000 chip (Agilent), and their concentration was calculated using the Qubit® fluorometer (Life Technologies). All samples were indexed, and multiplex sequencing was conducted on a HiSeq2500 instrument (Illumina, San Diego, CA, USA) to generate 60-base reads in single-end format. FastQ files for each sample were obtained using CASAVA v1.8 software (Illumina). Reads were aligned to the Ensembl reference genome and converted to reads per gene with the STAR software.

### Gene expression analysis

The DESeq2 package for R was applied to normalize gene counts and identify differentially expressed genes across the study conditions [47]. Gene set enrichment analysis (GSEA) was also used to infer enriched gene signatures from the Molecular Signatures Database (MSigDB) [48]. The normalized enrichment score (NES) was used to rank the enriched gene sets in each phenotype. A false discovery rate q-value (FDR *q*-value) was computed to estimate the probability that a gene set with a given NES represented a false positive finding. Over-representation analysis (ORA) was applied to determine the biological functions or processes enriched in the differentially expressed genes of each treatment condition using WebGestalt [49].

Normalized Gene expression data from PALOMA-2 (GSE133394) and PALOMA-3 (GSE128500) was obtained from the GEO repository. The R package ssGSEA2 (sample Gene set enrichment analysis 2) was used to compute the gene sets enrichment in each sample [50]. The following sets from the Human Molecular Signatures Database [51] were used: HALLMARK MTORC1 SIGNALING, HALLMARK G2M CHECKPOINT, HALLMARK FATTY ACID METABOLISM, HALLMARK HYPOXIA, HALLMARK E2F TARGETS, HALLMARK GLYCOLYSIS, HALLMARK OXIDATIVE PHOSPHORYLATION, HALLMARK IL2 STAT5 SIGNALING, HALLMARK PI3K AKT MTOR SIGNALING, HALLMARK MYC TARGETS V1 and HALLMARK MYC TARGETS V2. The cor.test function from R was used to evaluate the Pearson Correlation between *PRMT* genes expressed in PALOMA2/3 samples (*CARM1* and *PRMT1*) and pathway enrichment scores and expression levels of Palbociclib-resistance genes [52, 53].

### Targeted metabolomics by HPLC-MS/MS and FIA-MS/MS analysis

Intracellular metabolites were extracted from tumors frozen in dry-ice cooled isopentane with 85:15 EtOH:PBS buffer and then sonicated 3 times for 5 s each, submerged in liquid nitrogen for 30 s, and thawed at 95 °C twice. Then, extracts were centrifuged at 20,000 × *g* for 5 min at 4 °C, supernatants collected, and protein content determined. Cell media were collected at the beginning and the end of a 24-h incubation in exponential growth, and the cell number was determined for normalization purposes. Extracts from tumors and media were analyzed using the Absolute IDQ p180 kit (Biocrates Life Sciences AG, Innsbruck, Austria). Briefly, samples, calibration standards, and quality controls were dried under nitrogen gas flow at room temperature for 30 min. Next, 50 µl of 5% (v/v) phenylisothiocyanate (PITC) solution in 1:1:1 ethanol:water:pyridine solvent were added, incubated for 20 min at room temperature, and dried under nitrogen gas flow for 1 h. Then, the metabolites were resuspended in 300 µl of 5 mM ammonium acetate in methanol for 30 min with agitation. Samples were diluted 1:1 with Milli-Q water for tandem mass spectrometry coupled to high-performance liquid chromatography (HPLC-MS/MS) or diluted 1:10 with FIA mobile phase additive (provided by the kit) for flow injection analysis (FIA) coupled to MS/MS. Analyses were performed with an Agilent 1290 Infinity Ultra-High Performance Liquid Chromatography (UHPLC) system (Agilent, Santa Clara, CA, USA) coupled with an MS/MS Sciex Triple Quad 6500 (AB Sciex LLC, Framingham, MA, USA) using electrospray ionization (ESI) in positive mode and according to the manufacturer’s instructions. The chromatographic separation was performed on an Agilent ZORBAX Eclipse XDB C18 (Agilent, Santa Clara, CA, USA) column (3.5 μm particle size, 100 × 3.0 mm) using a gradient program at a constant flow rate of 500 µl/min. The oven temperature was maintained at 50 °C. HPLC mobile phases were 0.2% formic acid in water (solvent A) and 0.2% formic acid in acetonitrile (solvent B). The elution gradient was programmed starting at 100% of solvent A for 0.5 min, then decreasing the percentage of solvent A to 5% for 5 min, holding at 95% of solvent B for 1 min, and finally re-equilibrating the column at 100% of solvent A during 0.5 min and maintaining these final conditions for 2.5 min. On the other hand, the FIA mobile phase was methanol with FIA Mobile phase Additive provided by the kit. FIA gradient started at a flow rate of 30 µl/min for 1.6 min, then increased linearly until reaching 200 µl/min during 0.8 min, maintained for 0.4 min, and finally decreased to 30 µl/min in 0.2 min. Quantitation was achieved using multiple reaction monitoring scan mode.

Data analysis was performed using Analyst (AB Sciex LLC) and MetIDQ^TM^ (Biocrates Life Sciences AG) software. A total of 21 amino acids, 21 biogenic amines, 40 acylcarnitines, 90 glycerophospholipids, 15 sphingolipids, and hexose sugars were analyzed. Data were normalized by cell number for media samples and protein for tumor samples. Clustering, heatmaps, and statistical analysis were performed using MetaboAnalyst 5.0 [54].

### Cellular bioenergetics

The XFe24 Extracellular Flux Analyzer (Seahorse Bioscience, North Billerica, MA, USA) was used to measure the oxygen consumption, extracellular acidification, and proton efflux rates (OCR/ECAR/PER) in media immediately surrounding adherent cells cultured in an XFe24-well microplate (Seahorse Bioscience). One day before analysis, 7 × 10^4^ cells were plated in a monolayer in 100 µl, adding 150 µl of complete medium 4 h later, once cells were attached, and incubated at 37 °C and 5% CO_2_ overnight. Then, growth media were replaced by basal media (unbuffered DMEM, pH 7.4; Sigma-Aldrich) with or without glucose and incubated at 37 °C for 1 h without CO_2_. The sensor cartridge was hydrated with calibration solution (Seahorse Bioscience) overnight at 37 °C and loaded with the test reagents onto the Seahorse Analyzer to calibrate the sensors. Mitochondrial function and potential were analyzed by sequential injection of 1 µM oligomycin (ATP synthase inhibitor; Sigma-Aldrich), 0.6 µM Carbonyl Cyanide m-Chlorophenylhydrazone (CCCP, mitochondrial uncoupler; Sigma-Aldrich) together with 2 mM pyruvate to achieve maximal respiration, and 2 µM rotenone and antimycin A (mitochondrial complex I and III inhibitors, respectively; Sigma-Aldrich). Cells were counted at the end of the experiments to normalize the OCR, ECAR, and PER readings. Respiration, acidification, and ATP production rates were calculated following the Agilent Seahorse XF Technology instructions.

### Building condition-specific metabolic models

Condition-specific genome-scale metabolic models (GSMMs) for control and residual tumors from in vivo Palbociclib and Telaglenastat treatments were reconstructed from the human GSMM Recon3D [55]. Recon3D describes the metabolic potential of the human genome, yet in any given condition, only a subset of enzymes is expressed. Hence, we reconstructed condition-specific GSMMs containing only enzymes expressed in the study conditions. More in detail, enzymes with fragments per kilobase of exon per million mapped fragments (FPKM) under 1 in all conditions were removed, provided that their removal still enabled the models to produce 50% of optimal biomass as well as the synthesis or uptake of all amino acids and biogenic amines detected with the metabolomics assays. Additionally, enzymes that were expressed below 1 FPKM in a given treatment but not in control were also removed from the treatment-specific model if the difference in gene expression to the control was statistically significant (FDR adjusted *P*-value < 0.05).

### Quadratic multiomic metabolic transformation algorithm (qM^2^TA)

The quadratic multiomic metabolic transformation algorithm (qM^2^TA) models a metabolic transition between two states by maximizing the consistency between gene expression and metabolomics data and reaction fluxes (Fig. 5A, Supplementary Fig. 7). It allows the integration of both transcriptomics and metabolomics fold changes weighted by their statistical significance across both states and can identify key targets to revert the metabolic transformation.

To run qM^2^TA, we first computed the flux distribution in the control tumors condition (${v}_{i}^{{ref}}$, reference flux distribution) by applying the GIME3 algorithm [56]. Briefly, this algorithm consists of a flux minimization weighted by gene expression subject to achieving 95% of the maximum biomass production and producing all measured metabolites. Next, flux variability analysis [57] was used to identify the solution space within 99% of the GIME3 optimal solution. Finally, the resulting solution space was sampled using the Artificial Centering Hit-and-Run algorithm implemented into COBRApy [58, 59]. The average of these flux samples was used as the control/reference flux distribution.

Then, qM^2^TA optimization was used to simulate the metabolic transition upon treatment by maximizing the consistency between gene expression and metabolite concentration fold changes and simulated reaction flux fold changes relative to the control (Eq. 3). The optimization minimizes the difference between the resulting flux values and target flux (i.e., the product of reference flux value by fold change) for each differentially expressed metabolic gene or metabolite concentration. To give more weight to the features with more statistically significant differences, each feature (metabolite level or gene expression measure) was given a weight to the minimization inversely proportional to the P-value for the hypothesis that the fold change is different from 1. Additionally, flux variation was minimized for reactions not associated with differentially expressed genes or metabolites. The reference flux distribution scales both terms of the optimization to prevent a bias towards reactions with high reference flux.

3 $${min}\sum _{m\in {DExp}}\left({W}_{m}\sum _{i\,\in \,{R}_{m}}{\left(\frac{{v}_{i}^{{ref}}{{\cdot }}{{FC}}_{m}-{v}_{i}^{{res},q{M}^{2}{TA}}}{{v}_{i}^{{ref}}\left({{FC}}_{m}-1\right)}\right)}^{2}\right)+\sum _{i\in {Ru}}\frac{{\left({v}_{i}^{{ref}}-{v}_{i}^{{res},q{M}^{2}{TA}}\right)}^{2}}{{v}_{i}^{{ref}}}\,$$

$${W}_{{gm}}={LOG}10\left({p}_{{th}}\right)-{LOG}10\left({p}_{m}\right)$$

Subject to

$${\rm{s}}.\,{v}^{{res},q{M}^{2}{TA}}=0$$

$${{lb}\, < \,v}^{{res},q{M}^{2}{TA}}\, < \,{ub}$$

Where:

${DExp}$ is the list of differentially expressed genes and differentially abundant metabolites between the control and a target condition.

${W}_{m}$ is the weight given to measure $m$.

${R}_{m}$ are the reactions associated with measure $m$. For genes, this information is taken from the gene-reaction rules defined in Recon3D. Metabolomics measures are mapped to a sink reaction consuming the measured metabolite.

${v}_{i}^{{ref}}$ is the reference flux value (i.e., computed for the control condition) for reaction $i$.

${{FC}}_{m}$ is the gene expression or metabolite concentration fold change relative to the control.

${v}_{i}^{{res},q{M}^{2}{TA}}$ is the simulated flux value for reaction *i* in the condition of interest.

${Ru}$ are the reactions not associated with any differentially expressed genes.

$s$ is the stoichiometric matrix of the condition-specific GSMMs. The product of such matrix with ${v}^{q{M}^{2}{TA}}$ is set to 0 to define the steady-state constraint (i.e., inputs and outputs fluxes for each metabolite must be balanced).

*lb* and *ub* define reaction flux lower and upper bounds, respectively. For reactions identified as inactive when building condition-specific GSMMs, both *lb* and *ub* will be 0, preventing the reaction from carrying any flux. To simulate the effect of the glutaminase inhibitor, in the samples dosed with Telaglenastat or with the combination of Palbociclib and Telaglenastat, the maximum flux through glutaminase reaction (*ub*) was constrained to a maximum value of half the reference flux (${v}_{i}^{{ref}}$).

${W}_{g}$ is the weight given to each measured gene or metabolite.

${p}_{{th}}$ is the *P*-value threshold used to define a fold change in gene expression or metabolite levels as differentially expressed. The threshold was defined as 0.25 FDR-adjusted *P*-value.

${p}_{m}$ is the FDR-adjusted *P*-value for measure $m$ for the fold change between the condition under study and the control.

Comparing the flux values simulated in a given treatment ($${v}_{i}^{{res},q{M}^{2}{TA}})$$ to those in the control ($${v}_{i}^{{ref}}$$) can yield a unique insight into the metabolic reprogramming underlying the adaptation to the treatment-induced stress. Reactions were grouped into metabolic pathways to facilitate interpreting the results, and the fold changes of total flux values through each pathway relative to the control were computed. Reactions were assigned to metabolic pathways based on KEGG annotations for the genes catalyzing them.

Similar to MTA and rMTA [60, 61], qM^2^TA can identify gene targets that can disrupt a particular metabolic transformation, in this case, the metabolic adaptation to Palbociclib or Telaglenastat. The potential of each metabolic gene to impede such metabolic transformation was evaluated by systematically running qM^2^TA while reducing the maximum flux (*ub*) of reactions catalyzed by a given gene to 50% of the flux value in the control condition (${v}_{i}^{{ref}}$). This enabled testing each gene’s capacity to facilitate or disrupt the metabolic transformations associated with Palbociclib or Telaglenastat. This was complemented by testing the capacity of each gene knockdown (KD) to switch from the drug-adapted state to the control state [61]. The latter was achieved by running the minimization of metabolic adjustment (MOMA) algorithm while constraining the maximum flux through the reactions of a given gene to 50% of the flux value computed in the drug-adapted state (${v}_{i}^{{res},q{M}^{2}{TA}}$). The MOMA algorithm simulates the effect of perturbations by seeking the flux distribution that minimizes the variation in reaction fluxes compared to ${v}^{{res}}$ and subject to the constraints of the gene KD [62].

To evaluate the potential of each target, a transformation score (TS) [60, 61] was computed as follows:

4 $${TS}=\sum _{m\in {DE}}\left({W}_{m}\cdot {\rm{sign}}\left({\rm{Log}}\left({{FC}}_{m}\right)\right)\sum _{i\,\in \,{R}_{m}}{|v}_{i}^{{res}}|-{|v}_{i}^{{ref}}|\right)$$

Where,

${v}_{i}^{{res}}$ is the resulting flux distribution after either running qM^2^TA with no genes KD (wild type), running qM^2^TA with a gene KD, or running MOMA with gene KD.

A promising gene target would be any gene whose KD impairs the metabolic transformation from the control to the drug-adapted state while partially reversing the drug-adapted state towards the control state (Supplementary Fig. 7). This was measured with the difference between the base TS (i.e., computed when running qM^2^TA in the wild type) and the TS when running qM^2^TA (Eq. 5) and MOMA with a gene KD (Eq. 6).

5 $${{Dif}}_{g}^{q{M}^{2}{TA}}=\left({{TS}}_{q{M}^{2}{TA}}^{{base}}-{{TS}}_{q{M}^{2}{TA}}^{{g}_{{kd}}}\right)$$

6 $${{Dif}}_{g}^{{MOMA}}=\left(T{S}_{q{M}^{2}{TA}}^{{base}}-{{TS}}_{{MOMA}}^{{g}_{{kd}}}\right)$$

Where:

${{TS}}_{q{M}^{2}{TA}}^{{base}}$ is the TS score when running qM^2^TA in the wild type (i.e., without gene KD).

${{TS}}_{q{M}^{2}{TA}}^{{g}_{{kd}}}$ is the TS score when running qM^2^TA with gene *g* KD.

${{TS}}_{{MOMA}}^{{g}_{{kd}}}$ is the TS score when simulating the KD of target genes with MOMA starting from ${v}^{{res},{M}^{2}{TA}}$

Then, the target score ($${S}_{g})$$ for each gene is computed as follows:

7 $${S}_{g}=1000\frac{|{{Dif}}_{g}^{q{M}^{2}{TA}}\cdot {{Dif}}_{g}^{{MOMA}}|}{{\left({{TS}}_{q{M}^{2}{TA}}^{{base}}\right)}^{2}}\cdot \min \left({sign}\left({{Dif}}_{g}^{q{M}^{2}{TA}}\right),{sign}\left({{Dif}}_{g}^{{MOMA}}\right)\right)$$

### Statistical analysis

Statistical analysis was conducted using GraphPad Prism 7 software (GraphPad Software, San Diego, CA, USA). All data are expressed as mean ± standard deviation (SD) unless otherwise indicated. Shapiro–Wilk test was used to assess the normal distribution of experimental data, Levene’s test was employed to evaluate the homogeneity of variances, and Dixon’s Q-test was applied to identify outliers. Kruskal–Wallis test or one-way analysis of variance (ANOVA) and Tukey’s test were used for multiple comparisons between groups. Fisher’s least significant difference test was used to identify the groups that significantly differed from each other. Two-tailed independent sample Student’s *t* tests were used for comparisons between two conditions, and significant differences were indicated with asterisks. Differences were considered to be significant at *p* < 0.05 (*), *p* < 0.01 (**), and *p* < 0.001 (***). When specified, non-significant differences (*p* > 0.05) are shown as n.s. Differences between groups are indicated with different letters at p < 0.05. Groups with the same letter are not significantly different (*p* > 0.05). The sample size was based on prior studies in similar models to ensure reproducibility. Concerning gene expression analysis, the DESeq2 package for R was used to evaluate the differential expression between conditions using the Wald Test. The resulting *P*-values were corrected for multiple testing using the Benjamini-Hochberg function built into DESeq2.

## Results

### Metabolic adaptations of HCT116 cells to prolonged treatment with Palbociclib and Telaglenastat

**Fig. 1:**
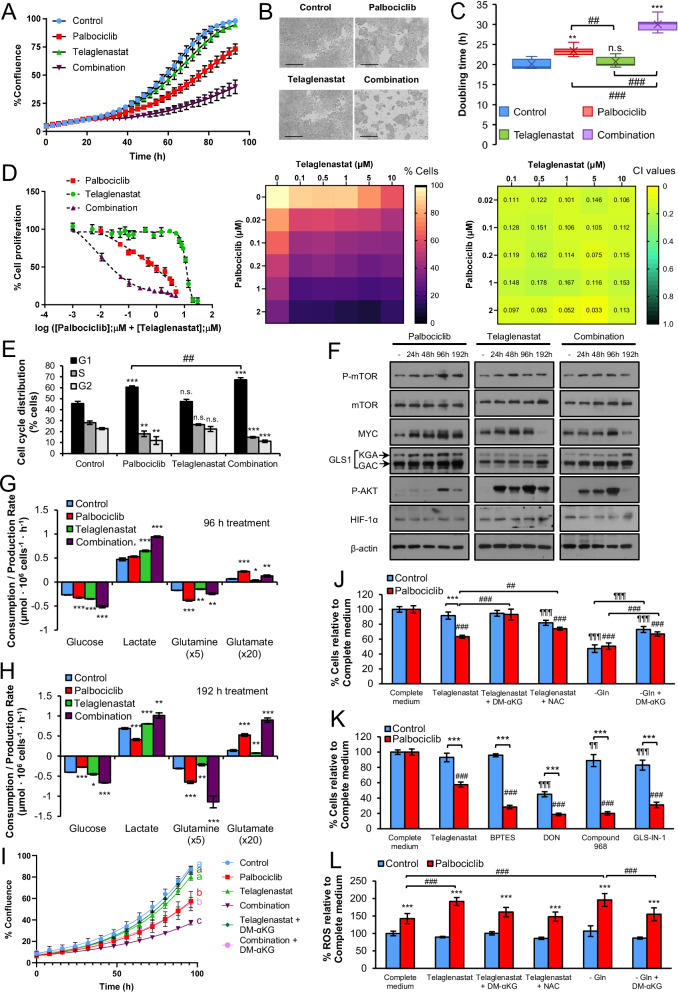
The antiproliferative effects of Palbociclib and Telaglenastat combination are synergistic and time-dependent. **A** Cell proliferation curves for HCT116 cells treated with Palbociclib, Telaglenastat, or their combination for 96 h. Confluence was monitored in an IncuCyte^®^ S3 (Sartorius) live-cell analysis system. Results are shown as mean ± SD with *n* = 6. **B** Representative IncuCyte^®^ S3 live-cell images in phase-contrast of HCT116 cells treated with Palbociclib, Telaglenastat, or their combination at 96 h. Cell confluence was monitored every 3 h. Scale bar = 400 μm. **C** Doubling time of HCT116 cells treated with Palbociclib, Telaglenastat, or their combination. Shown values are the mean ± SD of six independent experiments with six replicates each. **D** Cell proliferation assay. HCT116 cells were treated with increasing concentrations of Palbociclib, Telaglenastat, or their combination for 96 h, and cell quantification was assessed by Hoechst 33342 staining. Left, concentration-response curves generated by data fitting with a four-parameter equation. Center, dose-response cell proliferation matrix. Results are shown as the percentage of proliferation relative to untreated cells (mean ± SD for *n* = 6). Right, dose-response synergy matrix. The combination index (CI) values were obtained with CompuSyn software (ComboSyn, Inc., Paramus, NJ, USA), revealing a strong synergy (CI < 0.3) at each dose combination tested. **E** Cell cycle distribution in HCT116 cells treated with Palbociclib, Telaglenastat, or their combination for 96 h and determined by flow cytometry. **F** Effects of Palbociclib, Telaglenastat, or their combination on signaling pathways with time. P-mTOR, mTOR, MYC, GLS1, P-AKT, and HIF 1α protein levels were determined by Western blotting of total protein fractions using β-actin as a loading control. Comparative extracellular metabolic fluxes for HCT116 cells treated with Palbociclib, Telaglenastat, or their combination for (**G**) 96 h and (**H**) 192 h. Glucose and glutamine consumption and lactate and glutamate production rates were obtained after 24 h of incubation with fresh media and normalized to cell number. **I** Cell proliferation curves for HCT116 cells treated with Palbociclib, Telaglenastat, or their combination, in the presence or absence of dimethyl α-KG (DM-αKG) for 96 h. Confluence was monitored in the Olympus CM30 incubation monitoring system (Evident Corporation). Results are shown as mean ± SD with *n* = 6. Statistically significant differences between conditions are indicated with different letters. **J** Cell proliferation at 96 h following treatments with Palbociclib, Telaglenastat, or their combination in the presence or absence of dimethyl α-KG (DM-αKG), N-acetyl cysteine (NAC) or glutamine (Gln). Cell quantification was assessed by Hoechst 33342 staining. Results are shown as the percentage of proliferation relative to complete medium (mean ± SD for *n* = 6). **K** Cell proliferation at 96 h following treatments with Palbociclib, the glutaminase inhibitors Telaglenastat, BPTES, DON, Compound 968, Glutaminase-IN-1 (GLS-IN-1), or their combination with Palbociclib. Cell quantification was assessed by Hoechst 33342 staining. Results are shown as the percentage of proliferation relative to complete medium (mean ± SD for *n* = 6). **L** Determination of intracellular ROS levels after treatments with Palbociclib, Telaglenastat, or their combination in the presence or absence of dimethyl α-KG (DM-αKG), N-acetyl cysteine (NAC), or glutamine (Gln). ROS levels were determined by H_2_DCFDA staining. Results are shown as the percentage of proliferation relative to control cells in complete medium (mean ± SD for *n* = 6). Data information: Concentrations used were 2 µM for Palbociclib, 5 µM for Telaglenastat, and 5 µM for each glutaminase inhibitor. Shown values are mean ± SD for *n* = 3 (except otherwise indicated). Significance was determined by one-way ANOVA and two-tailed independent sample Student’s *t*-tests. Statistically significant differences between treated and control cells are indicated as *P* < 0.05 (*), *P* < 0.01 (**), and *P* < 0.001 (***), while differences between treatments are shown as *P* < 0.05 (#), *P* < 0.01 (##), and *P* < 0.001 (###) for Palbociclib-treated cells and *P* < 0.05 (¶), *P* < 0.01 (¶¶), and *P* < 0.001 (¶¶¶) for control cells.

We have previously shown that the combination of CDK4/6 and glutaminase inhibitors presents strong antiproliferative synergies in colon and breast cancer cells with no significant cytotoxic effects on non-malignant cells [36], predicting this combination as a promising therapeutic option in cancer treatment. However, the optimality of this drug combination and the underlying mechanisms driving its synergistic effects warrant further in-depth investigation. As a first approach, we characterized in vitro the antiproliferative effects of Palbociclib (PD0332991) (Supplementary Fig. 1A), alone and in combination with Telaglenastat (CB-839), a selective glutaminase inhibitor of both splice variants of glutaminase (KGA and GAC) with good oral bioavailability [63, 64], currently in phase II clinical trials (NCT04265534). As expected, Palbociclib imposed a pronounced antiproliferative effect on HCT116 cells (Fig. 1A, B and Supplementary Fig. 1B). However, the cell doubling time was only marginally increased (Fig. 1C), suggesting an early inhibition of cell division with a subsequent adaptation to this drug. In contrast, glutaminase inhibition alone had no impact on cell proliferation or duplication time. Also, the combination of Palbociclib and Telaglenastat presented strong synergistic antiproliferative effects over a broad dose range according to the CI equation of Chou and Talalay (CI < 0.3) (Fig. 1D and Supplementary Fig. 1C) on HCT116 colorectal cancer cells, as well as a reduction in anchorage-independent growth (Supplementary Fig. 1D) and clonogenic potential (Supplementary Fig. 1E). We also observed a synergistic effect on cell cycle arrest at the G1 phase for the combined condition (Fig. 1E) and a significant rise in cell doubling time compared to all groups and, consequently, a greater inhibitory effect on cell proliferation (Fig. 1A–C and Supplementary Fig. 1B). To control for cell type-specific biases, since HCT116 cells exhibit microsatellite instability (MSI) [65], we examined the synergistic antitumor effects of the combined treatment in microsatellite stable (MSS) colon cancer cell lines from different consensus molecular subtypes (CMS) [66], including SW403 (CMS2), HT29 (CMS3), and SW620 (CMS4) cell lines. Our findings demonstrate that the antiproliferative synergistic effects of the combined treatment are independent of the CMS and microsatellite stability status (Supplementary Fig. 1F–H), since the combination of Palbociclib and Telaglenastat synergistically inhibited the proliferation of all cell lines at all concentrations tested, suggesting that this combination may be effective across all CRC molecular profiles. Collectively, these results corroborate that glutaminase inhibition synergizes with Palbociclib to impair colon cancer cell proliferation in vitro.

To determine whether the cellular response triggered by the inhibitors is exposure-time dependent, we treated HCT116 cells with Palbociclib, Telaglenastat, and their combination for 24, 48, 96, and 192 h. By analyzing protein levels of central metabolism regulators, we observed that Palbociclib caused an activation of the mTOR, MYC, and AKT signaling pathways, which reached peak levels at 96 h of treatment (Fig. 1F). On the other hand, Telaglenastat induced an earlier activation of these pathways, as well as an upregulation of HIF-1α, while glutaminase protein levels were only increased at 192 h of treatment. Remarkably, the levels of MYC and P-AKT substantially decreased at 192 h in all cases, revealing a metabolic reprogramming dependent on the time of drug exposure. We also observed that the drug combination prevented an early activation of MYC compared to either treatment alone.

Next, we assessed the cellular consumption and production rates of the major carbon sources at 96 and 192 h of treatment (Supplementary Fig. 1I). In line with our previous results [36], cells subjected to a 96 h incubation with Palbociclib displayed enhanced rates of glucose and glutamine consumption and of lactate and glutamate production (Fig. 1G), which is also in agreement with the reported activation of mTOR, MYC, and AKT signaling pathways and increased protein levels of glutaminase (Fig. 1F). Notably, after 192 h of treatment, Palbociclib-treated cells shifted to a reduced glycolytic profile, while remaining highly glutaminolytic (Fig. 1H). On the other hand, Telaglenastat partially inhibited glutaminolysis and increased glucose consumption and lactate production, at both 96 and 192 h. Interestingly, the combined treatment elicited the greatest increase in glucose uptake and lactate release at both time points. The combination of inhibitors also prompted an increment in glutaminolytic flux at 96 h compared to control and Telaglenastat-treated conditions, with a further increase after 8 days of treatment, paralleled by an upregulation of GLS1 protein levels at 192 h (Fig. 1F). These observations suggest that a shift to glutaminolysis is a major metabolic compensatory mechanism to sustain cell survival under stress.

Having observed an enhanced glutamine consumption and glutaminolysis in Palbociclib-treated cells, we sought to elucidate the metabolic fate of glutamine in these cells. Metabolomic (HPLC-MS) analysis confirmed an increased oxidative activity through the TCA cycle, as evidenced by elevated intracellular TCA intermediates and related metabolites (Supplementary Fig. 1J). Notably, glutamine levels remained stable, suggesting that the increase in glutamine consumption reflects an enhanced TCA cycle activity rather than glutamine accumulation. This is in line with our previous findings [36] that demonstrated that CDK4/6 inhibition primarily directs glutamine towards α-ketoglutarate (α-KG) production to replenish the TCA cycle. We further validated the essentiality of glutaminolysis in Palbociclib-treated cells by demonstrating that the synergistic antiproliferative effects of the combination can be rescued by supplementing the medium with cell-permeable α-KG (dimethyl α-KG) (Fig. 1I, J and Supplementary Fig. 1K). This effect is consistent across ER^+^, HER2^-^ (MCF-7), ER^-^, HER2^+^ (SK-BR-3), and triple-negative (MDA-MB-231) breast cancer cell lines (Supplementary Figs. 1K, L). Importantly, HCT116 control cells were not affected by Telaglenastat concentrations that significantly reduced proliferation in Palbociclib-treated cells (Fig. 1I, J and Supplementary Fig. 1K).

To further validate that increased glutaminolysis drives Palbociclib resistance, we tested a panel of small molecule glutaminase inhibitors with diverse mechanisms of action, including the allosteric KGA inhibitor, BPTES, the glutamine analog, 6-Diazo-5-oxo-L-nor-Leucine (DON) that inhibits all glutamine-utilizing enzymes, the allosteric GAC inhibitor, Compound 968, and the conformational KGA inhibitor, Glutaminase-IN-1 (Fig. 1K). As expected, Palbociclib-treated cells exhibited sensitivity to all glutaminase inhibitors tested, while control cells were only highly sensitive to the broad-spectrum glutamine antagonist DON (Fig. 1K) and glutamine deprivation (Fig. 1J). This differential response indicates that the acquired sensitivity to glutaminase inhibitors in Palbociclib-treated cells can be explained specifically by their increased reliance on glutamine flux through the TCA cycle. Furthermore, long-term Palbociclib-resistant HCT116 cells, which displayed a 4-fold increase in Palbociclib IC_50_ values compared to parental HCT116 cells (Supplementary Fig. 1M), also exhibited significantly heightened sensitivity to all tested glutaminase inhibitors (Supplementary Fig. 1N). Therefore, long-term Palbociclib-resistant HCT116 cells showed greater dependence on glutaminolysis for proliferation than their parental counterparts. Interestingly, under glutamine deprivation conditions, supplementation with dimethyl α-KG only partially rescued proliferation in both control and Palbociclib-treated cells (Fig. 1J), indicating that non-anaplerotic functions of glutamine, such as protein and nucleotide synthesis, and redox homeostasis, are important for the proliferation of both cell types.

Given that enhanced glutaminolysis and OXPHOS can increase reactive oxygen species (ROS) production, we analyzed ROS levels after 96 h of incubation and observed that Palbociclib alone significantly augmented ROS production, while Telaglenastat further enhanced ROS levels only in Palbociclib-treated cells (Fig. 1L). In Palbociclib-treated cells, glutamine deprivation elevated ROS to levels comparable to those observed with the combination. This increment of ROS was prevented by both dimethyl α-KG and N-acetyl cysteine (NAC) in cells treated with the combination, which is in accordance with the observed rescue of cell proliferation under these conditions (Fig. 1J).

### Palbociclib and Telaglenastat combination has synergistic antiproliferative effects in vivo

**Fig. 2:**
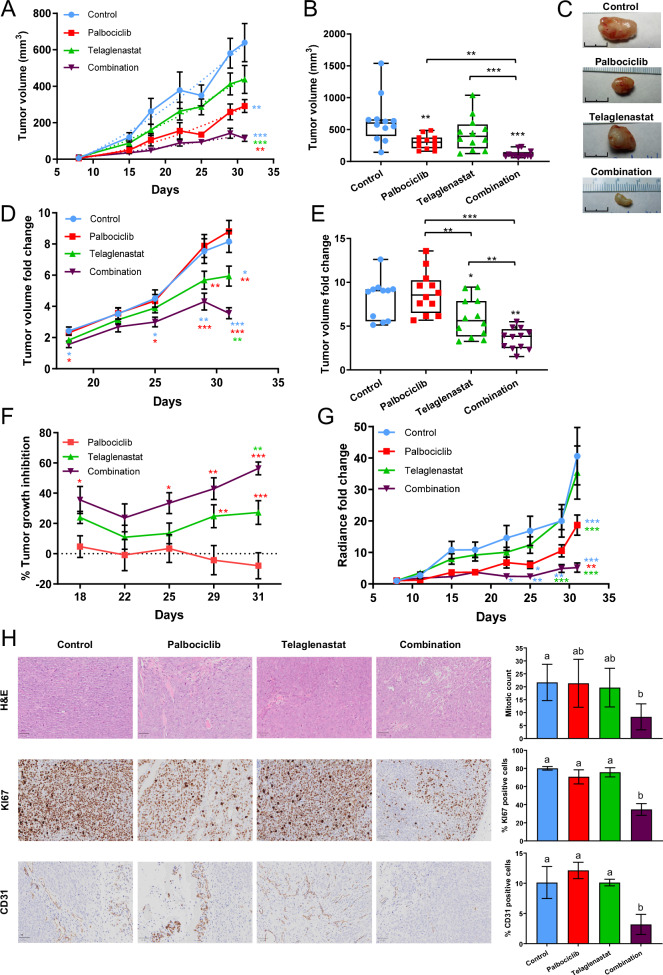
Palbociclib and Telaglenastat combined therapy significantly suppresses tumor growth in vivo. **A** Growth curve of HCT116 xenografts. After 1 week of tumor establishment, NOD-SCID mice were treated daily for 23 days with vehicle (Control), Palbociclib, Telaglenastat, or their combination, and tumor volume was measured with a Vernier caliper at the indicated days using the modified ellipsoid volume formula: Tumor volume = (length × width^2^) × π/6 (*n* = 12 per group). Data are presented as mean ± SEM. **B** Final volume measured at necropsy (*n* = 12 per group). Data are represented in a box and whiskers plot, with the whiskers representing the minimum and maximum values, all data points shown, and the median indicated. **C** Representative ex vivo images of solid tumors from each treatment group excised from NOD-SCID mice on day 31 after cell implantation. Scale bar = 1 cm. **D** Tumor volume fold change from day 18 (normalized to volume on day 15) in HCT116 subcutaneous colorectal cancer xenografts (*n* = 12 per group). Data are shown as mean ± SEM. **E** Tumor volume fold change at day 31 normalized to volume on day 15 (*n* = 12 per group). Data are represented in a box and whiskers plot, with the whiskers representing the minimum and maximum values, all data points shown, and the median indicated. **F** Bioluminescence intensity relative to the first measurement time point of photon radiance (day 8) in luciferase-expressing HCT116 xenografts (*n* = 12 per group). Data are shown as mean ± SEM. **G** Tumor growth inhibition (TGI) rates comparing the anti-tumor efficacy of the treatments from day 18 (*n* = 12 per group) calculated as TGI = (1 − (V_(T,t)_/V_(T,0)_)/(V_(C,t)_/V_(C,0)_)) × 100, where time 0 is the first measurement of tumor volume on day 15. Data are presented as mean ± SEM. **H** Representative histopathology images of human colon carcinoma xenografts generated from HCT116 cells; magnification ×200, scale bar = 50 μm. Top, Hematoxylin and Eosin (H & E) staining and mitotic figures quantification (mitotic count) expressed as the number of mitoses per 10 high-power fields (HPF), with HPF = ×400 overall magnification. Middle, KI67 immunohistochemical staining and quantification of the percentage of KI67 positive cells. Bottom, CD31 immunohistochemical staining and quantification of the percentage of CD31 positive cells. Shown values are mean ± SD for *n* = 3. Statistically significant differences between conditions are indicated with different letters. Data information: Concentrations used were 100 mg/kg/day p.o. for Palbociclib, 150 mg/kg/day p.o. for Telaglenastat, and 100 mg/kg/day p.o. Palbociclib + 150 mg/kg/day p.o. Telaglenastat for the combination. Shown values are mean ± SD for *n* = 12 (except where stated otherwise). Significance was determined by ANOVA and Tukey’s multiple comparisons test. Statistically significant differences between conditions are indicated as *P* < 0.05 (*), *P* < 0.01 (**), and *P* < 0.001 (***).

To validate our in vitro results, we investigated the effects of the combined administration of Palbociclib and Telaglenastat on the tumor growth of HCT116 cells, xenografted into NOD SCID mice. One week after HCT116 subcutaneous injection, mice were treated with either vehicle (Control), Palbociclib (100 mg/kg/day p.o.), Telaglenastat (150 mg/kg/day p.o.), or Palbociclib + Telaglenastat (100 mg/kg/day p.o. + 150 mg/kg/day p.o.) for 23 days (Supplementary Fig. 2A). Previous studies determined that these doses were non-toxic [63, 67]. Accordingly, mice tolerated all treatments without significant body weight loss (Supplementary Fig. 2B, C). Of note, animals gavaged with the combined treatment gained less weight than the other three groups (Supplementary Fig. 2D, E). Remarkably, while Palbociclib treatment alone induced a significant reduction in tumor volume compared to the control group (Fig. 2A–C), the tumor growth rate of both groups was equivalent (Fig. 2D, E). As a result, mice treated with Palbociclib alone did not display tumor growth inhibition (%TGI) compared to the control condition (Fig. 2F). These results suggest that inhibition of CDK4/6 has only an early impact on tumor cell proliferation and that surviving cells can overcome this inhibitory effect and subsequently restore their growth. On the other hand, treatment with Telaglenastat alone failed to achieve a statistically significant reduction of tumor volume (Fig. 2A–C). Nevertheless, at the end of the dosage period, tumors from mice treated with Telaglenastat exhibited a reduction in their growth rate (Fig. 2D, E) and about 20% of TGI (Fig. 2G), indicating a delayed effect of this treatment. Consistent with our in vitro studies, mice treated with the Palbociclib and Telaglenastat combination presented the smallest tumors (Fig. 2A–C) and the slowest tumor growth rates (Fig. 2D, E). These results were also confirmed by bioluminescence imaging and quantification of photon radiance (Fig. 2G and Supplementary Fig. 2F). In addition, we observed increasing TGI with time when Palbociclib was co-administered with Telaglenastat (Fig. 2F). Likewise, tumors from mice treated with the combined therapy presented the lowest proliferative activity as determined by mitotic and KI67 indexes, and also displayed reduced angiogenesis, as measured by CD31 immunohistochemistry (Fig. 2H and Supplementary Fig. 2G). In contrast, compared to the control group, tumors treated with Palbociclib or Telaglenastat alone did not present any changes in the levels of these markers. There were strong positive correlations between KI67 index and CD31^+^ cells (Pearson *r* = 0.828, *p* < 0.001), as well as between mitotic and KI67 indexes (Pearson *r* = 0.726, *p* < 0.01) or mitotic index and CD31^+^ cells (Pearson *r* = 0.607, *p* < 0.04) (Supplementary Figs. 2H–J). Collectively, our results reveal that the Palbociclib and Telaglenastat combined treatment can effectively forestall acquired resistance to Palbociclib and significantly improve the efficacy of either treatment alone in vivo.

### Metabolic features of residual tumors from Palbociclib and Telaglenastat treatments in vivo

Next, we studied the residual xenograft tumors at the end of the treatments by RNA sequencing (RNA-seq) and targeted metabolite profiling analyses. A total of 268 genes were differentially expressed in response to Palbociclib treatment (184 downregulated, 84 upregulated), 6028 genes in response to Telaglenastat (3290 downregulated, 2738 upregulated), and 4774 genes in response to the combined treatment (2527 downregulated, 2247 upregulated), compared to the control group (Supplementary Fig. 3A and Supplementary Table 1). Focusing on metabolic genes, 28, 580, and 500 genes were differentially expressed between the control group and tumors treated with Palbociclib, Telaglenastat, or their combination, respectively (Supplementary Fig. 3B and Supplementary Table 1). Notably, metabolism emerged as the biological process most impacted after treatment with Telaglenastat alone or in combination with Palbociclib (Supplementary Fig. 3C). Tumors subjected to the dual treatment presented 581 and 87 differentially expressed metabolic genes compared to Palbociclib and Telaglenastat single treatments, respectively, implying higher metabolic differences with Palbociclib than with Telaglenastat treatment (Supplementary Fig. 3D and Supplementary Table 1).

**Fig. 3:**
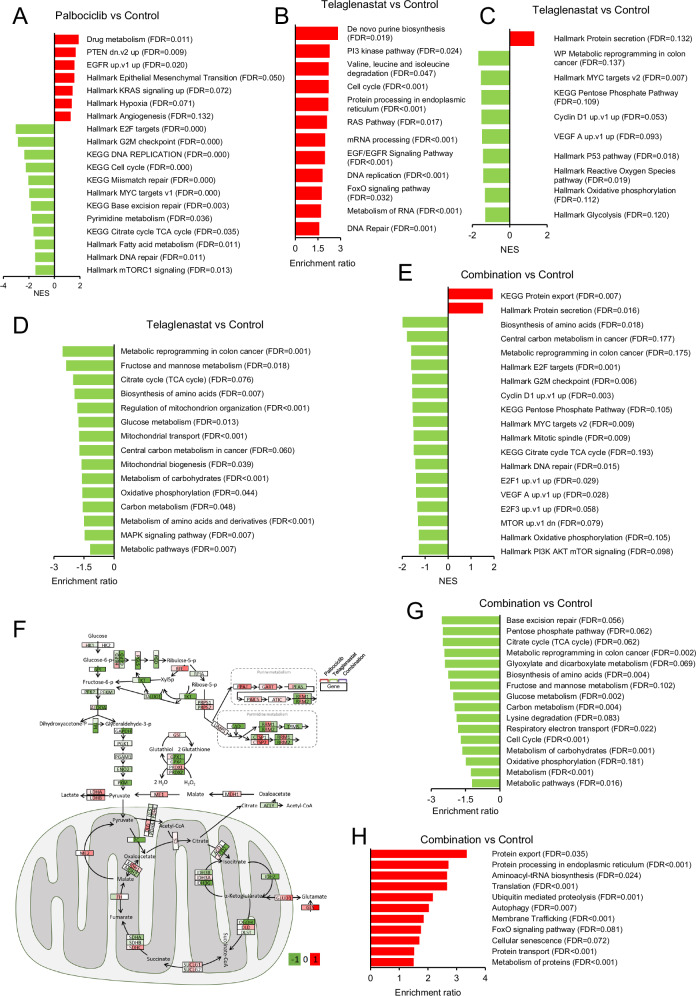
Transcriptomic analysis of Palbociclib, Telaglenastat, and their combination treatments in vivo. **A** Gene set enrichment analysis (GSEA) results of Palbociclib in vivo treatment compared to control. RNA-Seq was performed on tumors collected after resection and immediately frozen in liquid nitrogen or isopentane. The false discovery rates *q* values (FDR) are indicated for each gene set. Gene sets significantly enriched (FDR < 0.25) are ordered by NES. A positive normalized enrichment score (NES) value (bars in red) indicates enrichment in the Palbociclib treatment phenotype, while a negative NES (bars in green) indicates enrichment in the control phenotype. **B** Over-representation analysis (ORA) of upregulated gene sets in tumors treated with the Telaglenastat for 23 days (FDR < 0.25). Genes with FDR < 0.05 were used for the analysis. **C** GSEA results of Telaglenastat in vivo treatments compared to control. Gene sets significantly enriched (FDR < 0.25) are ordered by NES. A positive NES value indicates enrichment in the Telaglenastat treatment phenotype. **D** ORA of downregulated gene sets in tumors treated with Telaglenastat for 23 days (FDR < 0.25). Genes with FDR < 0.05 were used for the analysis. **E** GSEA results of Palbociclib and Telaglenastat in vivo combined treatment compared to control. Gene sets significantly enriched (FDR < 0.25) are ordered by NES. A positive NES value indicates enrichment in the combination treatment phenotype. **F** Schematic representation of the changes in gene expression of the central carbon metabolism caused by Palbociclib (left position in the gene label), Telaglenastat (central position), and their combination (right position) in vivo treatments. ORA of downregulated (**G**) or upregulated (**H**) gene sets in tumors treated with the combination of Palbociclib and Telaglenastat for 23 days (FDR < 0.25). Genes with FDR < 0.05 were used for the analysis. Data information: FDR false discovery rate, NES normalized enrichment score.

Gene set enrichment analysis (GSEA) [48] revealed that residual tumors from mice treated with Palbociclib alone displayed a positive enrichment for gene signatures related to drug metabolic mechanisms, KD of the PTEN tumor suppressor, activation of EGFR and KRAS signaling, epithelial-mesenchymal transition, angiogenesis, and hypoxia, and a negative correlation with gene sets associated with the cell cycle, MYC targets, pyrimidine, TCA cycle and fatty acid (FA) metabolism, and DNA replication and repair (Fig. 3A and Supplementary Figs. 4A, B and 5A). These results suggest an adaptation to Palbociclib treatment towards a more aggressive tumor phenotype that differs from the adaptive response previously reported after short in vitro treatments [36].

In contrast, residual tumors from mice treated with Telaglenastat alone presented an increased enrichment for gene sets involved in the cell cycle, DNA replication and repair, and oncogenic pathways such as RAS, EGF/EGFR, and PI3K (Fig. 3B and Supplementary Fig. 4C), while they were negatively enriched for metabolic gene sets and exhibited an enrichment in protein secretion hallmark genes (Fig. 3C, D and Supplementary Fig. 4D). The upregulation of cell cycle-related processes and oncogene-driven pathways in Telaglenastat-residual tumors is an unexpected outcome, which may explain the sustained proliferation observed in tumors treated with Telaglenastat alone.

The upregulation of metabolic pathways in tumors that survived Palbociclib treatment, and of cell cycle and oncogenic networks in those that survived Telaglenastat suggest complementary mechanisms of resistance between these two drugs. These observations also point to reciprocal vulnerabilities elicited by each drug. Supporting this hypothesis, analysis of residual tumors from mice treated with the Palbociclib and Telaglenastat combination revealed a systematic downregulation of gene sets associated with metabolism, cell cycle, *MYC* oncogene, and DNA repair (Fig. 3E), and of genes encoding enzymes of the central carbon metabolic pathways such as glycolysis, PPP, tricarboxylic acid (TCA) cycle, OXPHOS and FA metabolism (Fig. 3F and Supplementary Fig. 5B–F). ORA confirmed these observations by showing a global reduction in the enrichment score for metabolic pathways, cell cycle, and base excision repair (Fig. 3G and Supplementary Fig. 4E) and a positive enrichment for senescence-related processes (Fig. 3H and Supplementary Fig. 4F). The latter phenotype was further supported by a significant enrichment for gene signatures associated with protein secretion and export [68] and was in correspondence with the CellAge cellular senescence gene expression signature [69] (Supplementary Fig. 5G), indicating that the combined treatment triggers this anti-tumor mechanism leading to an irreversible arrest of cell division.

### Metabolomics point to novel vulnerabilities of tumor cells surviving Palbociclib and Telaglenastat treatment in vivo

**Fig. 4:**
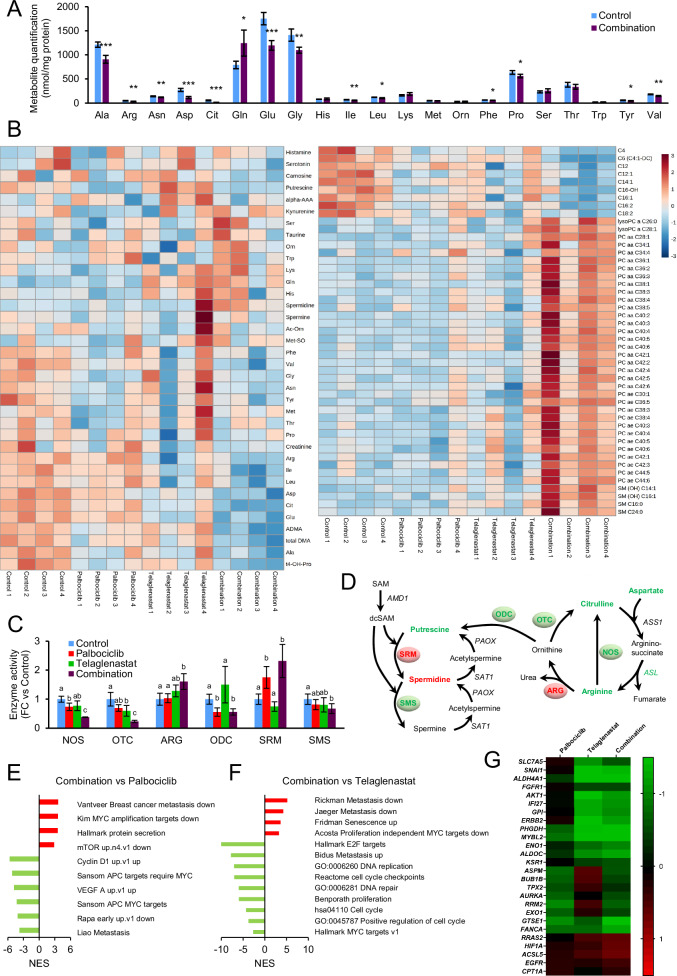
Metabolic characterization of in vivo tumors. **A** Quantification of amino acids in tumors treated with vehicle or the combination of Palbociclib and Telaglenastat using tandem mass spectrometry coupled to high-performance liquid chromatography (HPLC-MS/MS). Significance was determined by two-tailed independent sample Student’s *t*-tests. Statistically significant differences between treated and control cells are indicated as *P* < 0.05 (*), *P* < 0.01 (**), and *P* < 0.001 (***). **B** Heatmaps of metabolite quantification profiles from tumors treated with vehicle, Palbociclib, Telaglenastat, or the combination of Palbociclib and Telaglenastat obtained by tandem mass spectrometry coupled to high-performance liquid chromatography (HPLC-MS/MS) or flow injection analysis (FIA-MS/MS). For phosphatidylcholines (PC), the total number of carbon atoms and double bonds of the diacyl (aa) or acyl–alkyl (ae) groups is represented by Cx:y, where *x* indicates the number of carbons and y the number of double bonds. The same notation is used for describing the length and the number of double bonds in the acyl chain of acylcarnitines (**C**), lysophosphatidylcholines (lysoPC), sphingomyelins (SM) and hydroxylated sphingomyelins (SM (OH)). **C** Estimation of nitric oxide synthase (NOS), ornithine transcarbamylase (OTC), arginase (ARG), ornithine decarboxylase (ODC), spermidine synthase (SRM), and spermine synthase (SMS) enzyme activities relative to the control condition. Significance was determined by ANOVA and Tukey’s multiple comparisons test with *α* = 0.05. Statistically significant differences between conditions are indicated with different letters. **D** Schematic representation of the changes in metabolites, enzyme activities (bold), and gene expression (italics) of the polyamine metabolism and urea cycle caused by Palbociclib and Telaglenastat combined treatment. Gene set enrichment analysis (GSEA) results of Palbociclib and Telaglenastat in vivo combined treatment compared to Palbociclib (**E**) and Telaglenastat (**F**) individual treatments. RNA-Seq was performed on tumors collected after resection and immediately frozen in liquid nitrogen or isopentane. The normalized enrichment scores (NES) are indicated for each gene set (FDR *q* value < 0.05). A positive NES value (bars in red) indicates enrichment in the combination treatment phenotype, while a negative NES (bars in green) indicates enrichment in the individual treatment condition. **G** Heatmap of the expression of genes associated with Palbociclib resistance, as identified in the PALOMA-2/3 clinical trials, in tumors treated with Palbociclib, Telaglenastat, or the combination of Palbociclib and Telaglenastat, depicting the differentially expressed genes in tumors treated with the combination compared to the Control group. Data information: Ala alanine, Arg arginine, Asn asparagine, Asp aspartate, Cit citrulline, Gln glutamine, Glu glutamate, Gly glycine, His histidine, Ile isoleucine, Leu leucine, Lys lysine, Met methionine, Orn ornithine, Phe phenylalanine, Pro proline, Ser serine, Thr threonine, Trp tryptophan, Tyr tyrosine, Val valine, alpha-AAA α-aminoadipic acid, Ac-Orn acetylornithine, Met-SO methionine sulfoxide, ADMA asymmetric dimethylarginine, DMA dimethylarginine, t4-OH-Pro trans-4-hydroxyproline, SAM S-adenosylmethionine, AMD S-adenosylmethionine decarboxylase, dcSAM decarboxylated SAM, PAOX polyamine oxidase, SAT1 spermidine/spermine *N*^1^-acetyltransferase, ASS argininosuccinate synthase, ASL argininosuccinate lyase. Shown values are mean ± SD for *n* = 4.

Leveraging the transcriptomic analyses, which clearly indicated a major role for metabolic reprogramming as a key mechanism of resistance to Palbociclib, we conducted a targeted metabolomics profiling of residual tumors from mice treated with Palbociclib, Telaglenastat or their combination. Compared to control tumors, those subjected to the combined treatment exhibited significantly reduced concentrations of amino acids (alanine, arginine, asparagine, aspartate, citrulline, glutamate, glycine, isoleucine, leucine, phenylalanine, proline, tyrosine, and valine) and an accumulation of glutamine, a reflection of glutaminase inhibition (Fig. 4A). Interestingly, the reductions in amino acid and acylcarnitine levels were more pronounced in tumors subjected to dual treatment, as compared to Telaglenastat or Palbociclib alone (Fig. 4B and Supplementary Fig. 6A), in consonance with the reduced metabolism inferred from the transcriptomic analysis. Principal component analysis confirmed that the drug combination caused the greatest aminoacidic (Supplementary Fig. 6B) and lipidomic (Supplementary Fig. 6C) shifts.

Conversely, dual treatment induced high levels of phosphatidylcholines and sphingomyelins (Fig. 4B and Supplementary Fig. 6D, E). This may suggest that cells accumulate lipids as essential components of membranes but are unable to duplicate due to the alterations of DNA synthesis, resulting in membrane remodeling. Further, in tumors treated with the drug combination, estimation of enzyme activities through metabolite ratios [70] revealed a decrease in the activity of stearoyl CoA-desaturase (SCD) (Supplementary Fig. 6F), involved in cell migration and invasion [71], in line with a downregulation of genes involved in tumor invasion and metastasis [72]. Dual treatment was also associated with a downregulation of the urea cycle and of spermine synthase (SMS) in the polyamine pathway (Fig. 4C, D and Supplementary Fig. 6G), consistent with the observed downregulation of MYC target genes (Fig. 3A). The accumulation of spermidine (Supplementary Fig. 6G) is associated with autophagy induction, which suppresses proliferation and promotes apoptosis in cervical cancer cells [73], and could explain the enrichment observed in autophagic processes in tumors treated with the combination (Fig. 3C).

The combined treatment also elicited a downregulation of arginine methylation (Supplementary Fig. 6H) and protein arginine methyltransferases (PRMTs) (Supplementary Fig. 6I), which orchestrate epigenetic modifications, chromatin architecture, transcription, signaling, splicing, DNA damage response, and cell metabolism, and are identified as promising cancer therapeutic targets [74]. Interestingly, assessment of the transcriptomic data from the PALOMA-2 (NCT01740427) and PALOMA-3 (NCT01942135) clinical trials for Palbociclib [52] evidenced a significant positive correlation between the expression of both *PRMT1* and *CARM1* genes and the overexpression of genes in glycolysis, OXPHOS, hypoxia, PI3K/AKT/mTOR and MYC signaling pathways (Supplementary Fig. 6J and Supplementary Table 2), which are associated with the acquisition of therapeutic resistance and shorter progression-free survival in the PALOMA-2/3 trials. Likewise, *PRMT1* and *CARM1* were positively correlated with the expression of Palbociclib resistance genes, while they were negatively correlated with the sensitivity genes identified in the PALOMA-2/3 trials comparative biomarker analyses [52, 53] (Supplementary Table 3).

These observations suggest that residual tumor cells that survive Palbociclib therapy have acquired chemoresistance features through an epigenetic mechanism. Indeed, the combination of Palbociclib with the selective inhibitor of type I PRMTs, GSK3368715 [75], caused a synergistic reduction of cell proliferation in HCT116, SW403, HT29, and SW620 colorectal cancer cell lines over a wide dose range (Supplementary Fig. 6K–N), regardless of their CMS subtype or microsatellite stability status. Furthermore, long-term Palbociclib-resistant HCT116 cells exhibited increased sensitivity to the inhibition of type I PRMTs, with 5-fold lower IC_50_ values (Supplementary Fig. 6O). This indicates that resistance to Palbociclib may also be overcome by direct type I PRMT inhibition.

On the other hand, tumors subjected to the combined treatment displayed a downregulated expression of metastasis, cell cycle progression, and MYC and mTOR oncogenic pathways, as well as an increase in protein secretion and senescence pathways, as compared to single-drug treatments (Fig. 4E, F). Consistently, in tumors treated with the combination, the expression levels of Palbociclib resistance genes identified in PALOMA-2/3 trials [52, 53] and PEARL (NCT02028507) [76, 77] were significantly decreased (Fig. 4G and Supplementary Fig. 6P). Indeed, Palbociclib-resistant genes identified in the PALOMA-2/3 and PEARL trials were enriched in the genes significantly downregulated in HCT116 cells that persisted after the combined treatment, with Fisher exact test P-values of 0.00113 (Odds ratio = 3.06) and 0.00283 (Odds ratio = 2.98), respectively.

### Genome-scale metabolic modeling reveals that Telaglenastat is uniquely suited to counter the adaptive metabolic reprogramming induced by Palbociclib

**Fig. 5:**
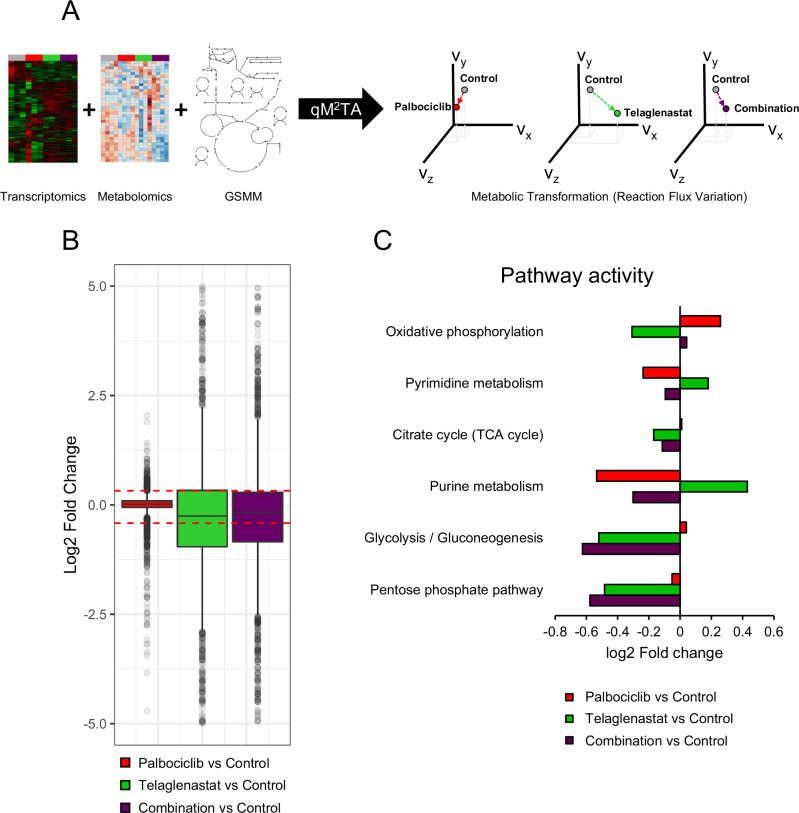
Quadratic Multiomic Metabolic Transformation Algorithm (qM^2^TA). **A** Schematic representation of qM^2^TA. qM^2^TA simulates the metabolic flux transition from the control condition to treatment with Palbociclib, Telaglenastat, or Palbociclib and Telaglenastat through the integration of transcriptomics and metabolomics into a genome-scale metabolic model (GSMM). A hypothetic 3D plot of three flux values (v_x_,v_y_,v_z_) and their transition with each treatment is provided. **B** Box plot of log2 fold changes in flux reaction values with each treatment relative to control. Dotted red lines indicate fold changes over 1.25 and under 0.75. **C** Variations in pathways of central carbon and oxidative metabolism with each treatment. Results are expressed as log2 fold changes for total pathway flux relative to the control.

To obtain a systems-wide perspective of the metabolic reprogramming underlying treatment with Palbociclib, Telaglenastat, or their combination, we integrated the transcriptomics and metabolomics data from the residual xenograft tumors in the framework of Recon3D. Recon3D is a genome-scale reconstruction of human metabolism, providing a mathematical representation of all the reactions and transport processes known in human cells and the enzymes and transmembrane carriers mediating them [55]. More in detail, we used the quadratic multiomic metabolic transformation algorithm (qM^2^TA) to impute the metabolic transformation underlying exposure to each drug and their combination by finding the intracellular metabolic flux rewiring most consistent with the transcriptomics and metabolomics measures (Fig. 5A and Supplementary Fig. 7A).

Treatment with Palbociclib alone resulted in 215 reactions with a flux value increase of 25% or more relative to control, whereas 239 had their flux reduced by 25% or more. Conversely, Telaglenastat alone and the combined treatment resulted in a larger number of reactions with reduced flux values relative to the control, 1461 and 1211, respectively, whereas only 726 and 701 reactions increased their flux values by 25% or more in such treatments (Supplementary Table 4). This suggests that Telaglenastat, alone or in combination, tends to have a largely inhibitory effect on metabolism, a notion also apparent by observing the global distribution of reaction flux values relative to the control (Fig. 5B).

Analysis of the flux through pathways of central carbon metabolism and OXPHOS, both key for cellular proliferation and the most relevant by the magnitude of carried flux, offers a more nuanced picture (Fig. 5C and Supplementary Table 4). For instance, Palbociclib treatment caused an increased flux through OXPHOS reactions and a decreased flux through purine and pyrimidine synthesis reactions. Indeed, activation of OXPHOS can likely be attributed to increased mTOR and MYC signaling, given that both promote mitochondrial biogenesis [78–80]. Conversely, Telaglenastat reduced the flux through the OXPHOS, glycolysis, and PPP while strongly upregulating purine and, to a lesser extent, pyrimidine metabolism. Finally, the combination of Palbociclib and Telaglenastat canceled out the metabolic readouts of each individual treatment, namely with no significant upregulation of OXPHOS and a reduced flux through the pathways of purine and pyrimidine synthesis, glycolysis, and PPP.

Upregulated pathways after treatment with Palbociclib or Telaglenastat likely reflect a process of metabolic reprogramming that allows cancer cells to adapt to and counter drug-induced stress. With this in mind, we used qM^2^TA to systematically search for potentially targetable enzymes or transmembrane carriers to prevent the metabolic adaptation to either Palbociclib or Telaglenastat (Supplementary Fig. 7B). We identified 327 and 280 potential targets to partially revert the metabolic adaptation underlying the Palbociclib or Telaglenastat treatments, respectively (Supplementary Table 4). Interestingly, glutaminase, the therapeutic target of Telaglenastat, was one of the targets against Palbociclib adaptation identified in this analysis. However, since qM^2^TA scores targets based solely on the direct effects of metabolic inhibitions on reaction fluxes, rather than downstream gene expression effects (see “Methods”), glutaminase effectivity as a target was underestimated by this analysis. Case in point, 112 targets inferred against Palbociclib adaptation, including many top-scoring targets, corresponded to genes that were significantly downregulated in response to Telaglenastat. Likewise, Palbociclib treatment downregulated 8 of the putative targets identified against Telaglenastat adaptation, including the second top-scoring putative target, ribonucleotide reductase. Indeed, putative targets against Telaglenastat-induced metabolic adaptation were significantly overrepresented (Fisher exact test *P*-value = 0.01319 and Odds ratio = 3.83) (Supplementary Table 5). These findings are in line with the patterns that emerged upon observation of flux changes through central carbon metabolism pathways (Fig. 5C). Indeed, they reinforce the notion that Palbociclib and Telaglenastat are uniquely suited to counter the metabolic reprogramming induced by the reciprocal drug, thus providing a mechanistic rationale for the high effectiveness of the drug combination observed in vitro and in vivo.

### Cells from residual tumors treated in vivo with Palbociclib and Telaglenastat maintain persistent drug-specific metabolic reprogramming ex vivo

Given that the effects of Palbociclib and Telaglenastat are time-dependent, we determined the potential persistence of their adaptive or resistant phenotypes after in vivo chemotherapy. To this end, we explanted 12 fresh residual tumors from mice treated for 23 days with vehicle, Palbociclib, Telaglenastat, or their combination, obtaining a total of 12 distinct two-dimensional monolayer cell cultures (three per in vivo treatment condition). Once established, we analyzed the glycolytic and glutaminolytic profiles of each cell line by incubation with complete media without any chemotherapeutic pressure.

**Fig. 6:**
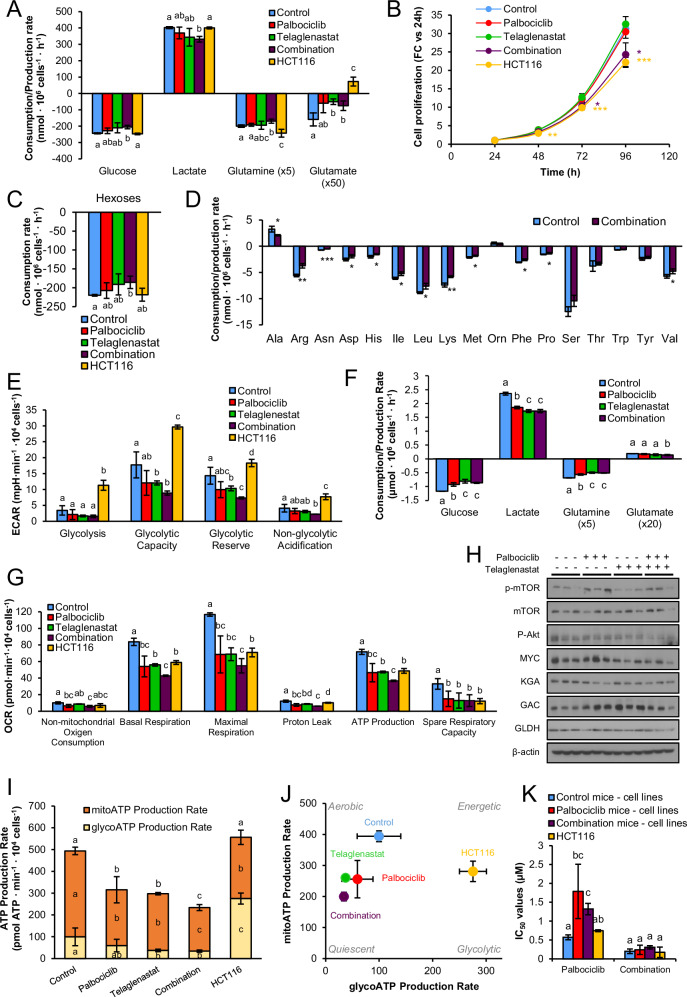
Metabolic characterization of cell lines derived from HCT116 xenografts. Twelve cell lines were obtained from HCT116 tumors from mice that had been treated daily with vehicle, Palbociclib, Telaglenastat, or the combination of Palbociclib and Telaglenastat for 23 days (three cell lines per condition). All cell lines were grown in the absence of chemotherapeutics. **A** Comparative extracellular metabolic fluxes. Glucose and glutamine consumption and lactate and glutamate production rates were assessed after 24 h of incubation with fresh media and normalized to cell number. **B** Cell proliferation curves. Results are shown as fold change of cell proliferation relative to the measurement at 24 h (mean ± SD of *n* = 6). Significance was determined by one-way ANOVA and Tukey’s multiple comparisons test. Statistically significant differences between cells derived from tumors that had been treated with the combination and cells obtained from control tumors are indicated as *P* < 0.05 (*). **C** Pool of hexoses consumption rate determined by targeted metabolomics after 24 h of incubation with fresh media and normalized to cell number. **D** Amino acid consumption and production rates were measured after 24 h of incubation with fresh media and normalized to cell number. Significance was determined by a two-tailed independent sample Student’s *t* test. Statistically significant differences between treated and control cells are indicated as *P* < 0.05 (*), *P* < 0.01 (**), and *P* < 0.001 (***). **E** Quantification of extracellular acidification rate (ECAR) for glycolysis, glycolytic capacity, glycolytic reserve, and non-glycolytic acidification. Cells were cultured in the absence of glucose, and sequential injections of glucose and oligomycin were applied. Data are normalized to cell number and represented as the mean of three independent cell lines for each condition ± SD with *n* = 5. **F** Spheroids comparative extracellular metabolic fluxes. Spheroids were grown in non-adherend conditions for each cell line, and glucose and glutamine consumption and lactate and glutamate production rates were assessed after 96 h of incubation with fresh media and normalized to cell number. **G** Quantification of oxygen consumption rates (OCR), following sequential injections of oligomycin (1 µM), CCCP (600 nM), and antimycin A (2 µM) and rotenone (2 µM), for non-mitochondrial oxygen consumption, basal respiration, maximal respiration, non-ATP-linked oxygen consumption (proton leak), ATP production-associated respiration, and spare respiratory capacity. Data are normalized to cell number and shown as the mean of three independent cell lines for each condition ± SD with *n* = 5. **H** Western blotting analysis of total protein fractions for the cell lines generated from tumors using β-actin as a loading control. Each lane corresponds to a different cell line. **I** Quantification of the ATP production rate from glycolysis (glycoATP) and mitochondrial oxidative phosphorylation (mitoATP) and their contribution to the total cellular ATP production rate. Data are normalized to cell number and displayed as the mean of three independent cell lines for each condition ± SD with *n* = 5. **J** Energetic map exhibiting the distribution of mitochondrial ATP (mitoATP) production rate vs. glycolytic ATP (glycoATP) production rate. Cells derived from tumors treated with the combination of Palbociclib and Telaglenastat presented the most quiescent metabolism. Values are expressed as the mean of three independent cell lines for each condition ± SD with *n* = 5. **K** IC_50_ values for Palbociclib and the combination of Palbociclib and Telaglenastat in cell lines derived from residual tumors from mice that had been treated with these inhibitors in vivo. Bars represent the mean of the IC_50_ values of three independent cell lines for each condition ± SD with *n* = 6. Data information: Ala alanine, Arg arginine, Asn asparagine, Asp aspartate, Cit citrulline, Gln glutamine, Glu glutamate, Gly glycine, His histidine, Ile isoleucine, Leu leucine, Lys lysine, Met methionine, Orn ornithine, Phe phenylalanine, Pro proline, Ser serine, Thr threonine, Trp tryptophan, Tyr tyrosine, Val valine. Significance was determined by one-way ANOVA and Tukey’s multiple comparisons test with *α* = 0.05. Statistically significant differences between conditions are represented with different letters. Data are shown as the mean of three independent cell lines for each condition ± SD with *n* = 3 (except otherwise indicated).

We found that cells derived from tumors that had been treated with the drug combination exhibited a decrease in the metabolic fluxes of glucose, glutamine, lactate, and glutamate (Fig. 6A), together with a reduction in proliferation and doubling time (Fig. 6B and Supplementary Fig. 8A) without affecting cell cycle distribution (Supplementary Fig. 8B). Likewise, the overall consumption rate of hexoses was only significantly reduced in the condition resulting from tumors treated with Palbociclib and Telaglenastat (Fig. 6C). In addition, we assessed the amino acid exchange fluxes and found that alanine production, and arginine, asparagine, aspartate, histidine, isoleucine, leucine, lysine, methionine, phenylalanine, proline, and valine consumption rates were diminished in cells from tumors that had been dosed with the combination therapy (Fig. 6D and Supplementary Fig. 8C). To further confirm the lower glycolytic rates observed, we measured in real-time the extracellular acidification rate (ECAR), essentially a sign of lactate production from glycolysis with a small contribution from the CO_2_ produced in other reactions, while sequentially adding glucose and oligomycin to cells cultured in a medium without glucose. As expected, cells derived from Palbociclib plus Telaglenastat-treated residual tumors portrayed a significant reduction in the glycolytic capacity, glycolytic reserve, and non-glycolytic acidification (Fig. 6E). Then, we confirmed that the metabolic downregulation caused by the long-term double treatment and reported in vivo and 2D ex vivo cultures was also maintained in spheroid cultures (Fig. 6F).

Considering that the TCA cycle and OXPHOS were among the gene sets downregulated in vivo by the treatments (Fig. 3), we performed a pharmacological profiling of the mitochondrial respiratory function by combining the ATP synthase inhibitor oligomycin, the protonophoric uncoupler CCCP, the complex III inhibitor antimycin A and the complex I inhibitor rotenone, while measuring the oxygen consumption rate (OCR). This analysis revealed that cells explanted from residual tumors from mice treated with the drug combination presented reduced non-mitochondrial oxygen consumption, basal respiration, maximal respiration, non-ATP linked oxygen consumption (proton leak), ATP production-associated respiration, and spare respiratory capacity, depicting a global reduction in mitochondrial respiratory capacity (Fig. 6G), consistent with the observed lower consumption rates of glucose and amino acids and the decrease of MYC, p-AKT, KGA and GLDH (glutamate dehydrogenase) protein levels (Fig. 6H). Moreover, transcriptomic analysis revealed that OXPHOS, one of the main pathways enriched during metastatic seeding and chemoresistance [81–83], was downregulated in cells derived from the combination therapy tumors compared to those obtained from control or single-agent treated tumors (Supplementary Fig. 8D).

### Combined Palbociclib and Telaglenastat treatment induces a less aggressive phenotype without the acquisition of resistance

Next, to compare the cellular function and energy demands of the surviving cancer cells after each chemotherapeutic treatment, we measured the ATP production rate ex vivo as a more accurate approach to assess the energetic cellular state than determining the total intracellular levels of ATP, which are maintained under steady-state conditions [84].

We observed that cells from residual tumors from mice that were treated with the combined therapy displayed the lowest rate of mitochondrial, glycolytic, and total ATP production, while the reduction in both single-agent treatments was less pronounced (Fig. 6I). Similarly, the contribution of mitochondria to oxygen consumption and proton production was significantly lower in cells previously treated in vivo with the combination (Supplementary Fig. 8E, F), consistent with a quiescent phenotype (Fig. 6J). Of note, xenograft-explanted cells presented a shift from glycolytic to mitochondrial metabolism compared to in vitro HCT116 cells not passaged in mice, which had an ATP rate index of 1 (Fig. 6I and Supplementary Fig. 8G). These results agree with the observed reduction in intracellular glutathione in cells derived from tumors treated with the combination or Telaglenastat alone (Supplementary Fig. 8H), considering that this antioxidant molecule is synthesized through two ATP-requiring steps from glutamate.

To complement this analysis, we performed GSEA on transcriptomics data, which showed that cells explanted from residual tumors from mice subjected to the combined therapy exhibited a downregulation of genes involved in the cell cycle machinery, MYC signaling, metastasis, OXPHOS, and electron transport chain, as compared to cells derived from tumors treated with Palbociclib alone (Supplementary Fig. 8I). These cells also exhibited a downregulation of genes regulated by oncogenes such as MYC, VEGF, and cyclin D1 and associated with mitochondria and amino acid metabolism when compared with cells from tumors treated with Telaglenastat alone (Supplementary Fig. 8J), revealing a persistent reduction in the malignant potential of surviving cells that had been exposed to combined therapy in vivo, as compared to those subjected to a single agent.

Finally, to examine whether the xenograft-derived cell lines had shifted their sensitivity profiles after in vivo exposure to the drugs, we exposed the cells to increasing doses of each drug or the combination for 96 h and determined IC_50_ values. While the effect of Telaglenastat on cell proliferation was similar in all cases, regardless of in vivo drug exposure (Supplementary Fig. 8K), in vivo exposure to Palbociclib alone or in combination with Telaglenastat conferred explanted tumor cells with a 2-fold increase in resistance to Palbociclib alone (Fig. 6K). Remarkably, the same cells were at least as sensitive as control HCT116 cells to the combination of Palbociclib and Telaglenastat (Fig. 6K). Moreover, the combination caused in vitro synergistic antiproliferative effects in the cell lines derived from tumors subjected to the combined therapy in vivo (Supplementary Table 6).

Together, these data demonstrate that colon cancer cells that survive to the long-term Palbociclib and Telaglenastat combined treatment display impaired glycolytic metabolism, mitochondrial respiration, and ATP production, as well as mitigated oncogenic pathways, resulting in persistent and less aggressive tumor cell phenotypes.

## Discussion

Targeting cell cycle through cyclin-dependent kinases 4 and 6 (CDK4/6) inhibition (CDK4/6i) is an established strategy in the treatment of several cancer types [3]. However, few cancer types are sensitive to CDK4/6i’s when used as single agents [31, 85]. Furthermore, sensitive cancers invariably acquire resistance to CDK4/6 inhibition [86], such that CDK4/6i’s are only efficacious in combination with drugs that target other pathways on which cancer cells rely to escape CDK4/6 dependency [87, 88]. Numerous pre-clinical studies and clinical trials are currently exploring the therapeutic potential of combining CDK4/6 inhibitors with endocrine therapies (e.g. aromatase inhibitors, ER antagonists), immunotherapies (e.g. antibodies to PD1/PD-L1), oncogenic kinase inhibitors (e.g. receptor tyrosine kinase-PI3K-AKT-mTOR signaling, RAS, BRAF, MEK, IGF-1R, ALK inhibitors), and other targeted or chemotherapeutic agents (e.g. autophagy, PARP inhibitors) in solid tumors [3].

Metabolic reprogramming is a relevant cell-autonomous mechanism through which tumor cells change their energetic dependencies to alternative pathways in order to bypass the selective pressures exerted by a given class of antineoplastic drugs [89, 90]. Such metabolic shifts may reveal vulnerabilities that may be targeted through combination therapies to prevent and overcome chemoresistance [82, 91]. Our previous work identified the inhibition of glutaminase as a metabolism-based strategy to combine with CDK4/6 targeting therapy [36], a combination subsequently corroborated as effective to overcome CDK4/6i resistance in esophageal squamous cell carcinoma [92] and lung cancer [93]. Of interest, upregulation of glutaminolysis as an adaptive mechanism is not limited as a survival response to CDK4/6i’s, having also been observed in response to other chemotherapeutic pressures, such as PARP inhibitors in breast cancer cells [94]. Here, we have undertaken an in-depth, unbiased metabolomics approach to assess in greater detail the metabolic reprogramming undergone by HCT116 colorectal cancer cells exposed to either the CDK4/6i Palbociclib, the glutaminase inhibitor Telaglenastat, or their combination.

First, we observed that the metabolic reprogramming undergone by cancer cells after treatment with either agent alone is time-dependent. Consistently, tumors in mice dosed with Palbociclib alone had the same growth rate as the control tumors despite exhibiting a significant reduction in their volume, suggesting that Palbociclib activity is also time-dependent in vivo and that, after the initial antiproliferative effect, surviving tumor cells acquire the capacity to overcome this inhibition and restore their proliferative rate. Telaglenastat did not exert any significant effect on tumor growth in xenotransplanted mice. In contrast, the synergistic antiproliferative effect of the dual treatment was time-independent and effective both in vitro and in vivo. As such, the combined administration of Palbociclib and Telaglenastat was strongly synergistic for the inhibition of tumor growth, proliferative index, and angiogenesis.

Gene set enrichment analysis confirmed that tumors subjected to the double therapy exhibited a downregulation in metastasis, cell cycle progression, and MYC and mTOR oncogenic pathways compared to control and single treatments. Lending translational significance to these observations, tumors from mice subjected to dual therapy presented a decrease in the expression of Palbociclib-resistance genes identified in the PALOMA-2/3 and PEARL clinical trials assessing biomarkers of sensitivity and resistance to Palbociclib in ER^+^ HER^−^ breast cancer [52, 53, 76, 77]. Of particular interest, the treatment of HCT116 tumor-bearing mice with Palbociclib and Telaglenastat provoked the downregulation of arginine methylation and PRMTs. Expression of PRMTs correlates with the activation of resistance pathways associated with lower progression-free survival [52] and is upregulated through MYC and mTORC1 activation [95], suggesting that Palbociclib-surviving cells might have acquired chemoresistance through an epigenetic mechanism.

The drug-specific metabolic reprogramming of residual cancer cells after in vivo treatments was maintained in ex vivo cultures for many cell generations, in the absence of any chemotherapeutic selective pressure, also suggestive of a heritable epigenetic mechanism underlying the persistent metabolic reprogramming. Specifically, cells derived from tumors treated with the Palbociclib and Telaglenastat combination presented a general decrease in central carbon metabolic fluxes in conjunction with an overall reduction in mitochondrial respiration, as well as in mitochondrial, glycolytic, and total ATP production. Notably, these cells displayed an impoverishment in oncogenic pathways compared to cells from tumors subjected to Palbociclib or Telaglenastat alone, indicating a less aggressive phenotype. Importantly, residual tumor cells from in vivo dual treatment were sensitive to the double treatment ex vivo. In contrast, residual tumor cells from in vivo treatment with Palbociclib as a single agent were resistant to this drug ex vivo. This indicates that (i) Palbociclib treatment readily elicits a resistant phenotype both in vitro and in vivo, (ii) the combination of Palbociclib with Telaglenastat disables Palbociclib-induced resistance mechanisms in vitro and in vivo and (iii) the disabling of resistance mechanisms by this drug combination is heritable through many cell generations.

By applying the quadratic multiomic metabolic transformation algorithm (qM^2^TA), we integrated metabolite and enzyme transcript levels to infer genome-scale metabolic rewiring upon treatment with Palbociclib, Telaglenastat, or their combination in vivo. From this analysis, it emerged that Palbociclib treatment resulted in increased flux through OXPHOS, attributable to increased mTOR and MYC signaling [78–80], and decreased flux through reactions involved in nucleotide synthesis. OXPHOS is often upregulated in cancer stem cells and drug-resistant tumors [81, 83] and our observations are in line with recent findings describing that the treatment of metastatic breast cancer with Palbociclib elicits increased OXPHOS as a resistance mechanism that can be countered pharmacologically [82]. In contrast to Palbociclib, Telaglenastat treatment caused a decreased flux through OXPHOS, glycolysis, and the PPP, while increasing flux through nucleotide metabolism. Notably, nucleotide synthesis, PPP, glycolysis, and TCA play a key role in the synthesis of biomass building blocks needed for proliferation and are commonly upregulated in cancer compared to healthy tissue [96]. Hence, the combination of Palbociclib and Telaglenastat maintained the benefits of each individual treatment while sparing the upregulation of OXPHOS as a resistance mechanism and reducing flux through nucleotide synthesis, glycolysis, and PPP. Furthermore, many markers of metabolic adaptation to Palbociclib were downregulated by Telaglenastat and vice versa. Taken together, our analysis indicates that the upregulation of OXPHOS through glutaminolysis is the most relevant mechanism of resistance to CDK4/6 inhibition in our cell model. As such, inhibition of glutaminolysis through Telaglenastat, or other selective GLS1 inhibitors, is an optimal strategy to overcome resistance to Palbociclib in cancer cells.

## Conclusions

In summary, we have delineated a major metabolic mechanism explaining resistance to Palbociclib in a colorectal cancer model, namely glutaminolysis-driven OXPHOS, and shown that the combination of a selective CDK4/6 inhibitor with a selective glutaminase inhibitor optimally resensitizes cancer cells to CDK4/6 inhibition through many cell generations. We believe that these findings warrant a proposal to use this drug combination in clinical settings.

## Supplementary information

Supplementary Figures

Supplementary Figure and Table Legends

Supplementary Table 1

Supplementary Table 2

Supplementary Table 3

Supplementary Table 4

Supplementary Table 5

Supplementary Table 6

## Data Availability

RNA-Seq data were deposited into the Gene Expression Omnibus database under accession number GSE245085 and are available at the following URL: https://www.ncbi.nlm.nih.gov/geo/query/acc.cgi?acc=GSE245085. All other data generated in this study are available upon request from the corresponding authors.

## Code availability

The functions and scripts needed to run GIME3 and qM^2^TA are available on GitHub at the following URL: https://github.com/cfoguet/cobrafunctions/tree/dev_python_3.10.
