# Supplementary material for: Glutaminase as a metabolic target of choice to counter acquired resistance to Palbociclib by colorectal cancer cells
Source: Oncogene. 2025 Jul 22;44(36):3386–406. doi: 10.1038/s41388-025-03495-w (PMC12399431; doi:10.1038/s41388-025-03495-w)

Supplementary Figure 1

A

Palbociclib (PD0332991) (*CAS* 571190-30-2)

Telaglenastat (CB-839) (*CAS* 1439399-58-2)

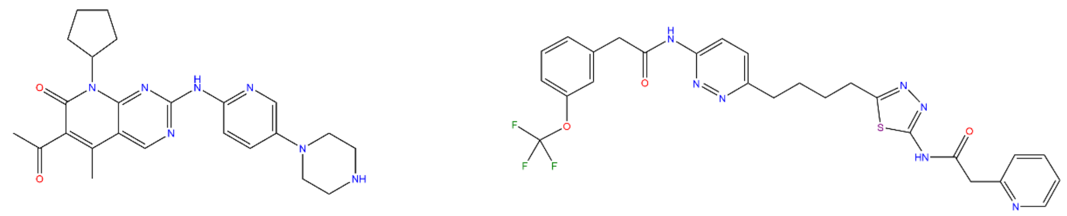

B

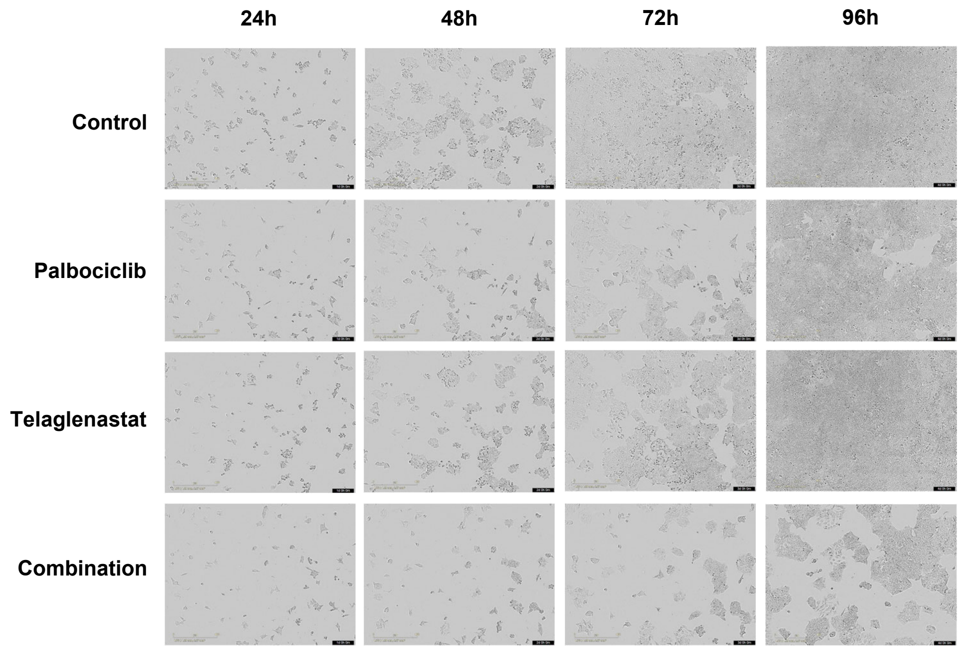

C

Combination Index (CI) equation of Chou and Talalay and CompuSyn software

CI value	Agonistic effect
<0.10	Very strong synergism
0.10–0.30	Strong synergism
0.30–0.70	Synergism
0.70–0.90	Moderate to slight synergism
0.90–1.10	Nearly additive
1.10–1.45	Slight to moderate antagonism
1.45–3.30	Antagonism
>3.30	Strong to very strong antagonism

[Palbociclib] μM	[Telaglenastat] μM	Viability	SD	CI Value
0.01	0.001	63.3	5.1	***0.030***
0.05	0.003	36.5	2.1	***0.034***
0.1	0.005	28.8	4.5	***0.042***
0.25	0.01	22.0	2.4	***0.065***
0.5	0.05	21.0	0.7	***0.120***
0.75	0.1	17.8	1.3	***0.137***
1	0.5	18.7	3.7	***0.199***
1.5	1.0	15.7	1.0	***0.224***
2	1.5	13.5	1.1	***0.235***
3	2.0	11.9	0.2	***0.290***

CI < 0.3

D

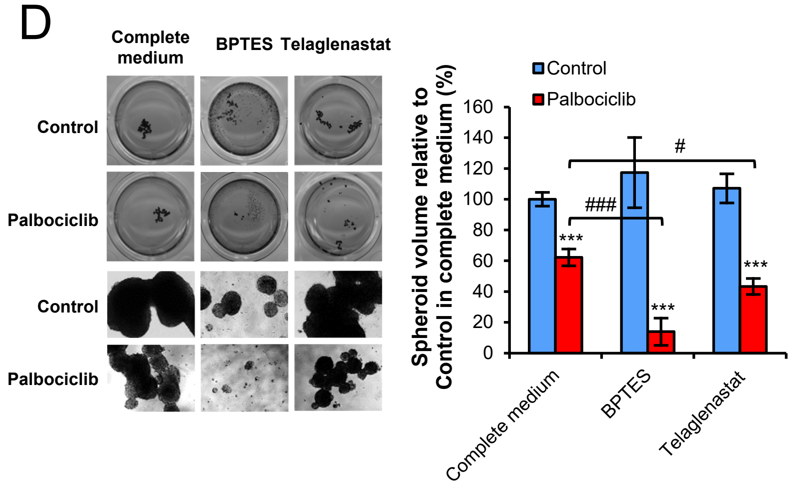

E

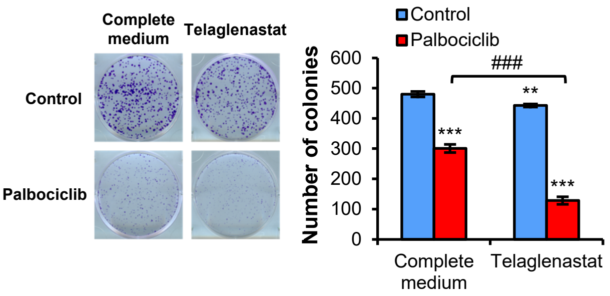

Supplementary Figure 1

F

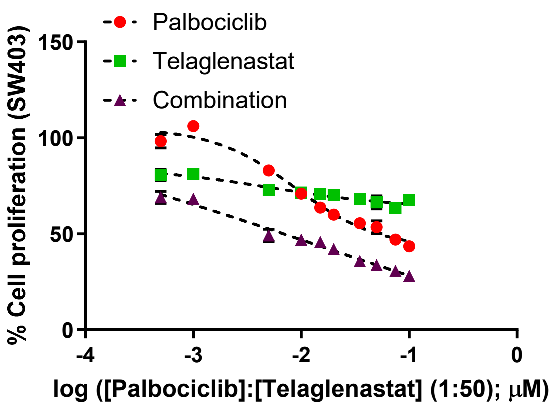

[Palbociclib] µM	[Telaglenastat] µM	Viability	SD	CI Value
0.0005	0.025	69.1	3.2	***0.055***
0.001	0.05	68.2	2.2	***0.098***
0.005	0.25	49.2	3.3	***0.086***
0.01	0.5	46.9	2.5	***0.145***
0.015	0.75	45.7	1.8	***0.201***
0.02	1	42.1	2.7	***0.205***
0.035	1.75	35.8	1.4	***0.223***
0.05	2.5	33.7	1.6	***0.269***
0.075	3.75	30.7	1.7	***0.315***
0.1	5	28.1	0.8	***0.336***

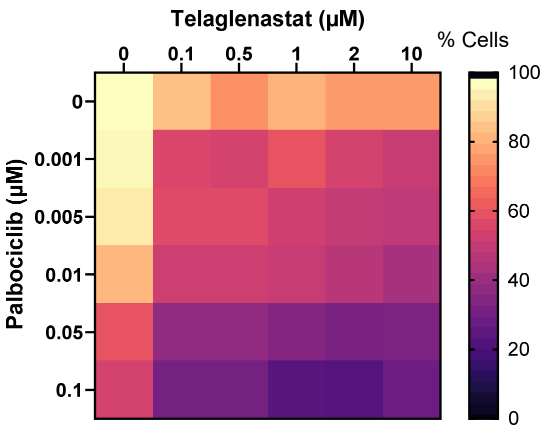

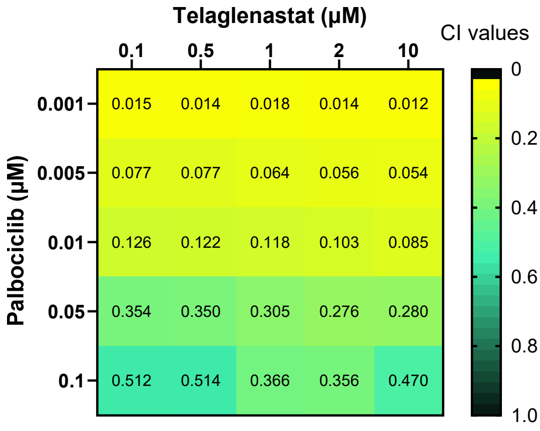

G

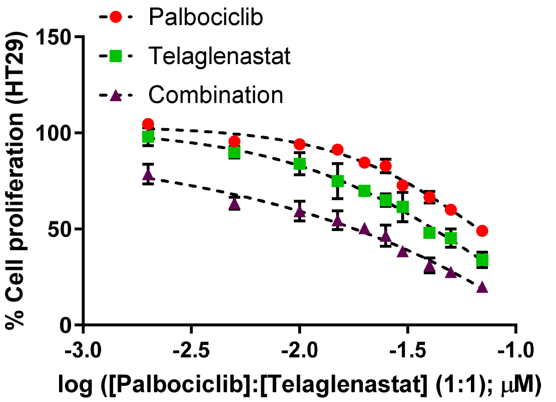

[Palbociclib] µM	[Telaglenastat] µM	Viability	SD	CI Value
0.002	0.002	78.5	5.2	***0.230***
0.005	0.005	63.4	3.1	***0.291***
0.01	0.01	59.3	5.1	***0.496***
0.015	0.015	54.6	4.9	***0.622***
0.02	0.02	50.4	1.7	***0.711***
0.025	0.025	46.6	5.5	***0.772***
0.03	0.03	38.4	2.9	***0.680***
0.04	0.04	31.0	3.9	***0.670***
0.05	0.05	27.6	1.9	***0.720***
0.07	0.07	19.9	2.6	***0.682***

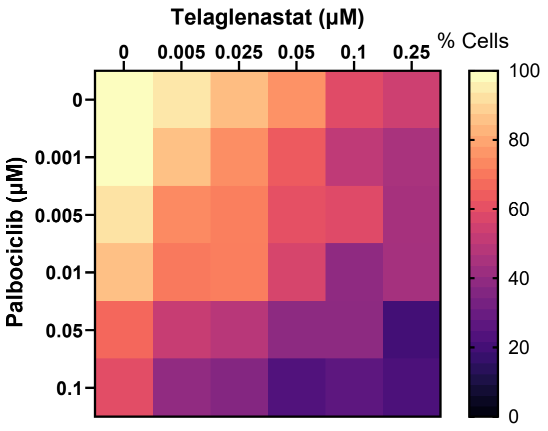

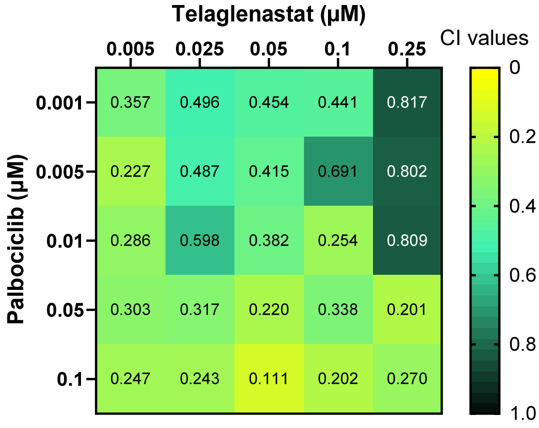

Supplementary Figure 1

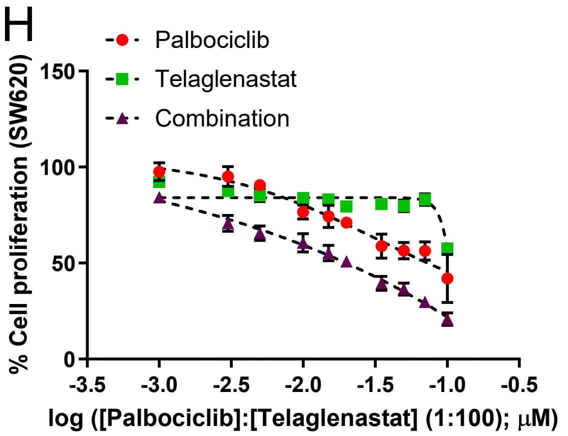

[Palbociclib] µM	[Telaglenastat] µM	Viability	SD	CI Value
0.001	0.1	84.2	1.6	***0.210***
0.003	0.3	70.7	4.2	***0.154***
0.005	0.5	65.6	3.8	***0.184***
0.01	1	60.6	4.8	***0.277***
0.015	1.5	55.2	4.0	***0.315***
0.02	2	50.8	2.3	***0.338***
0.035	3.5	39.4	3.6	***0.344***
0.05	5	36.4	3.1	***0.422***
0.07	7	29.7	2.4	***0.417***
0.1	10	20.8	3.2	***0.345***

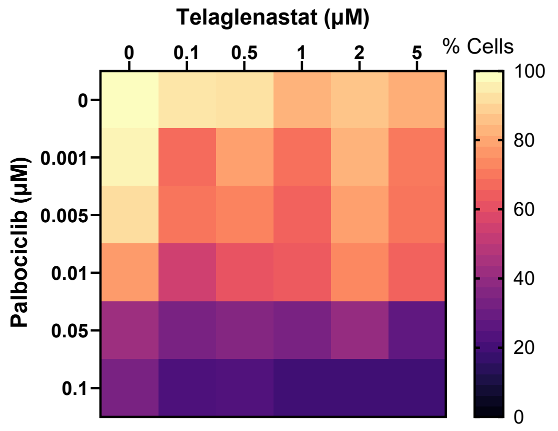

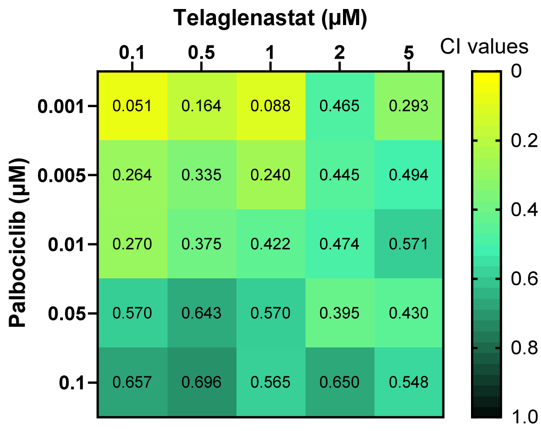

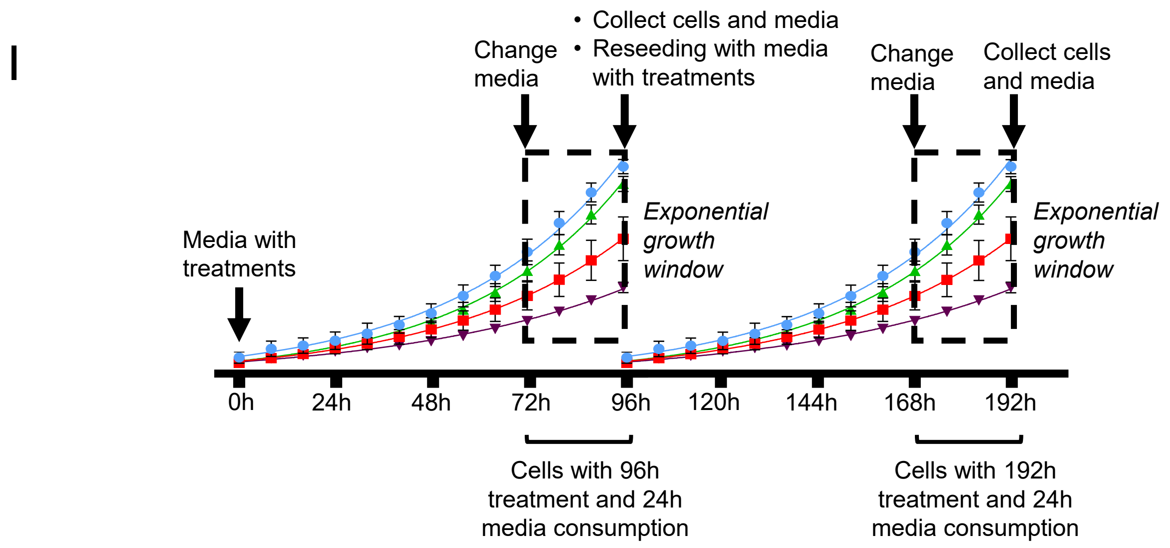

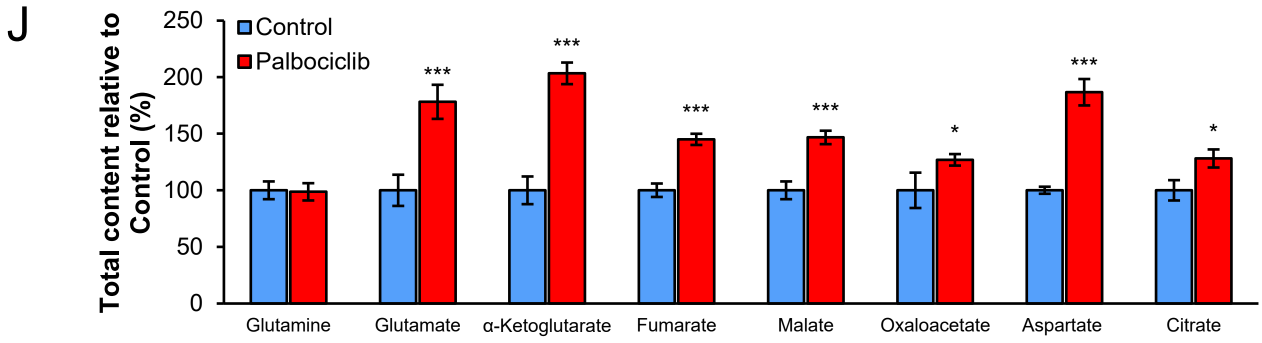

Supplementary Figure 1

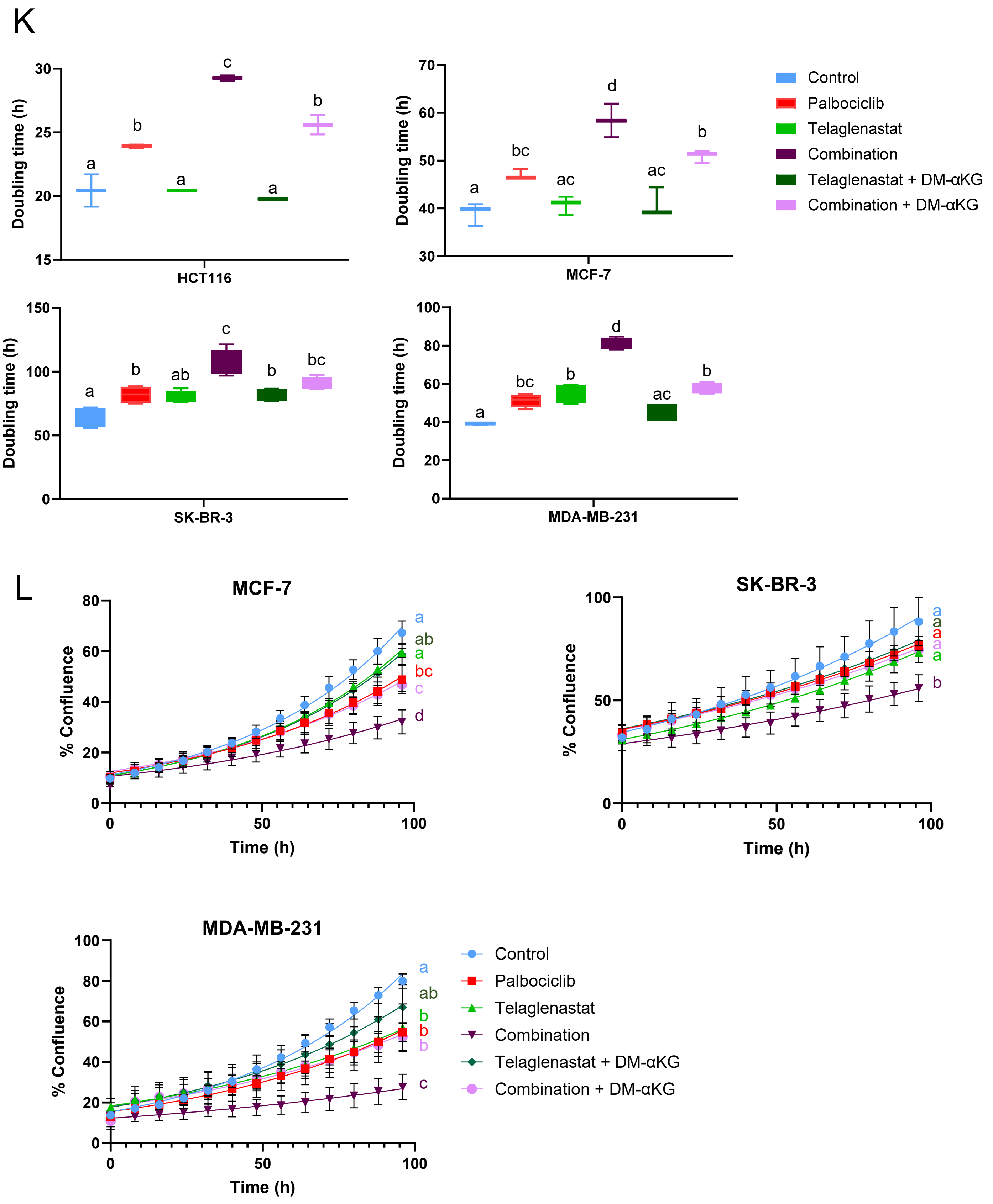

Supplementary Figure 1

M

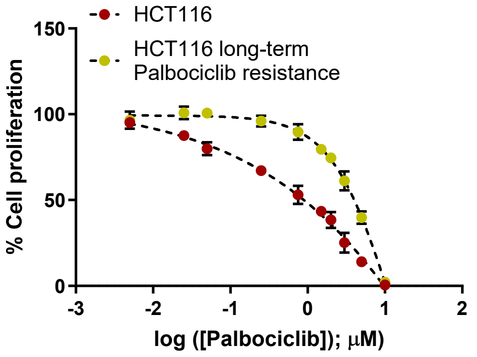

N

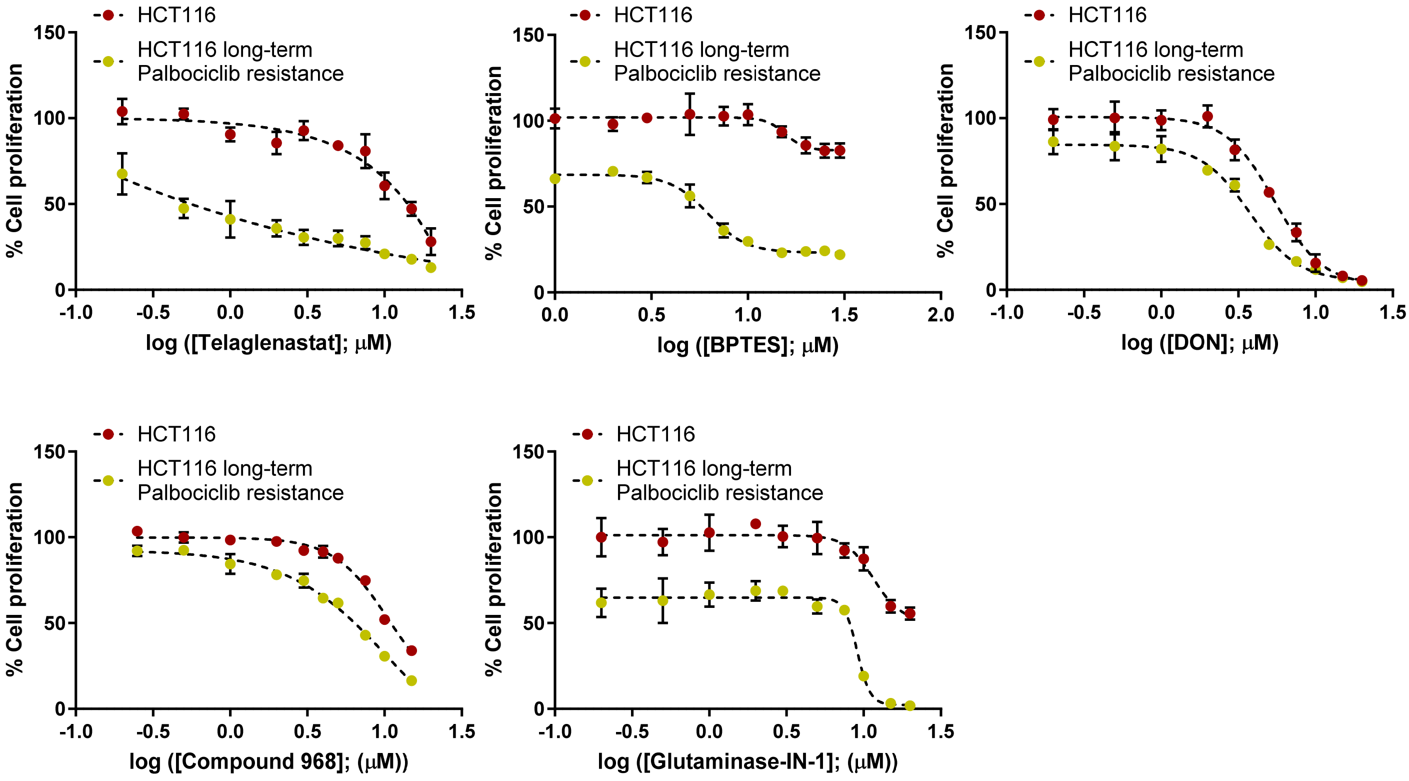

Supplementary Figure 2

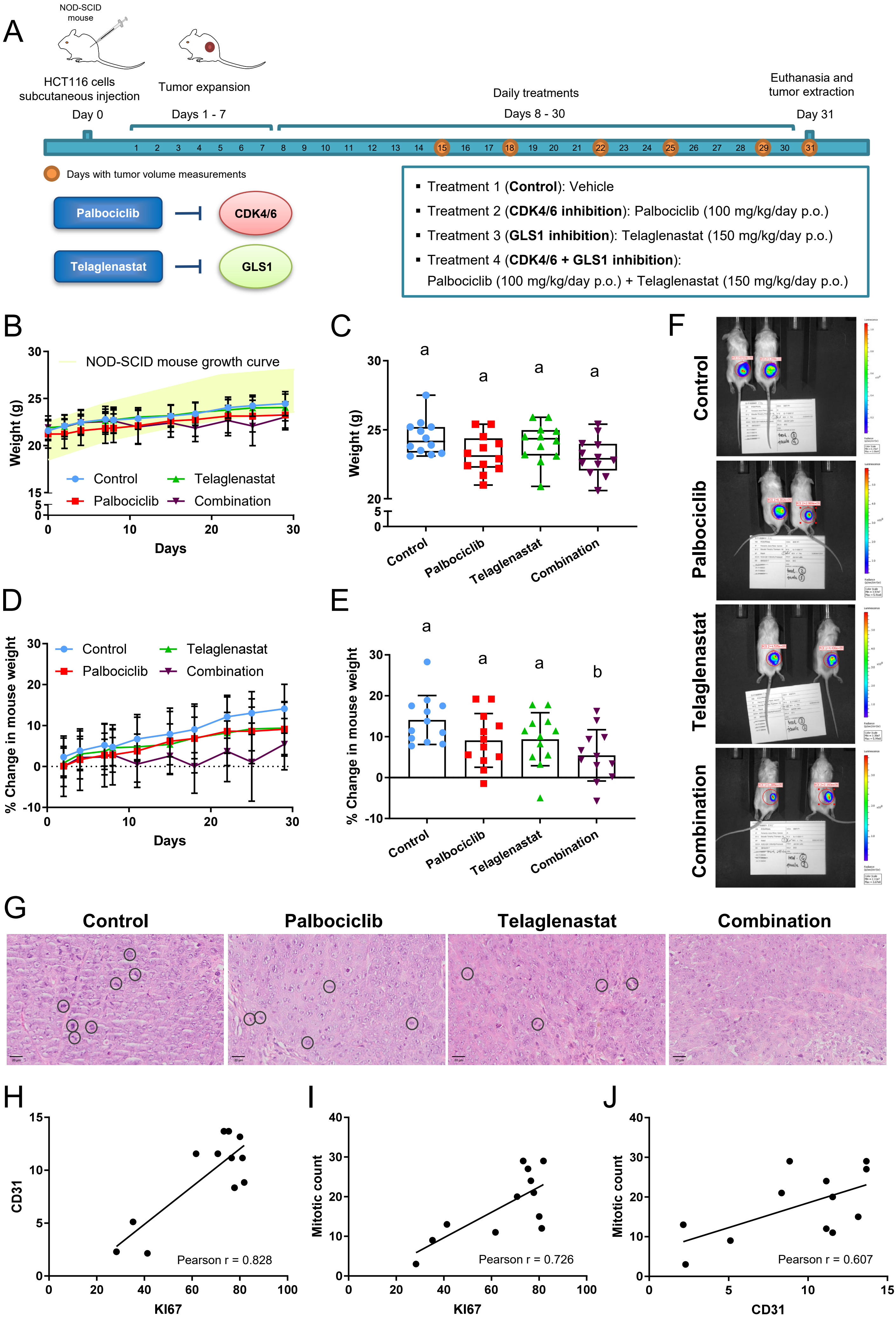

Supplementary Figure 3

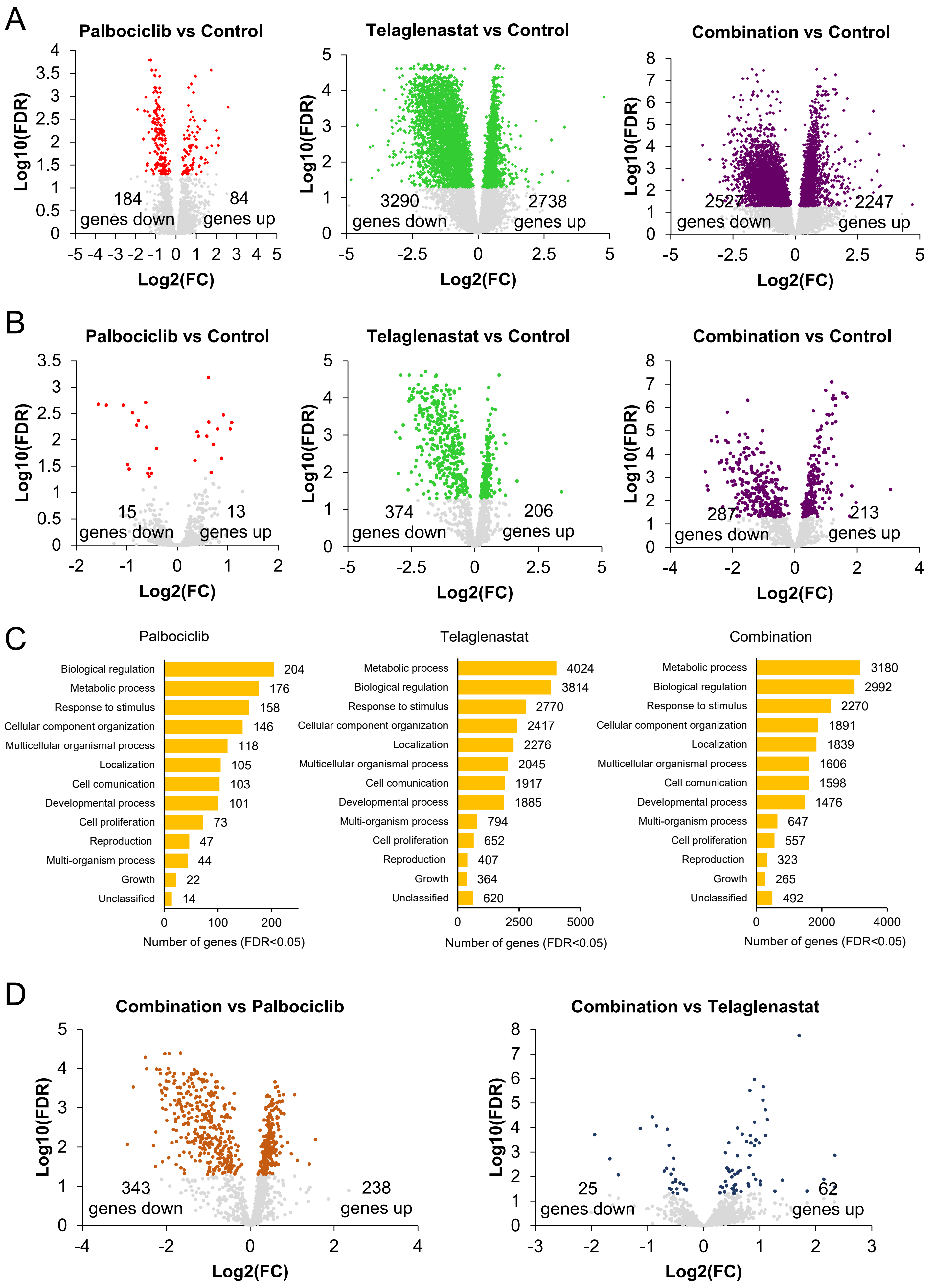

Supplementary Figure 4

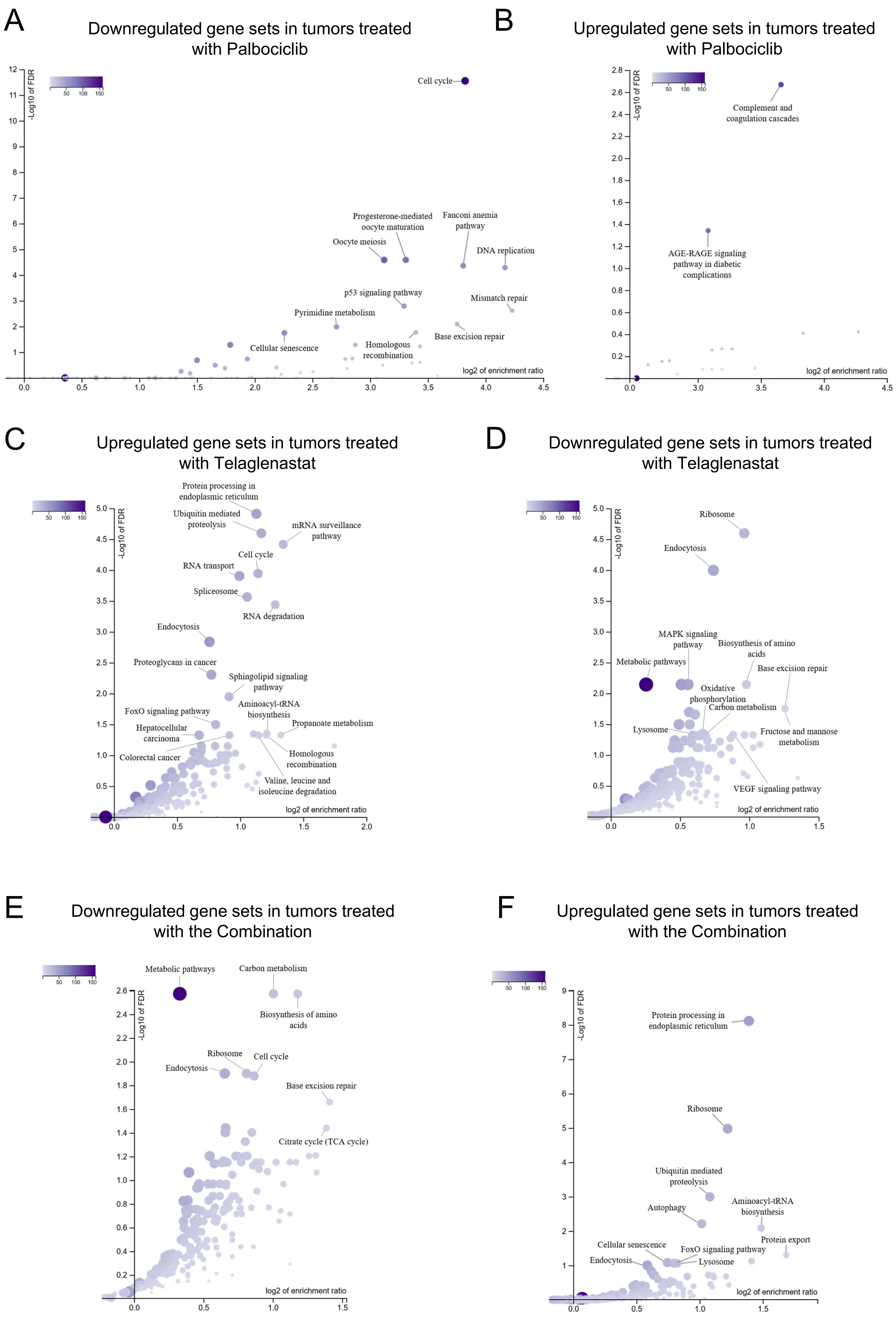

Supplementary Figure 5

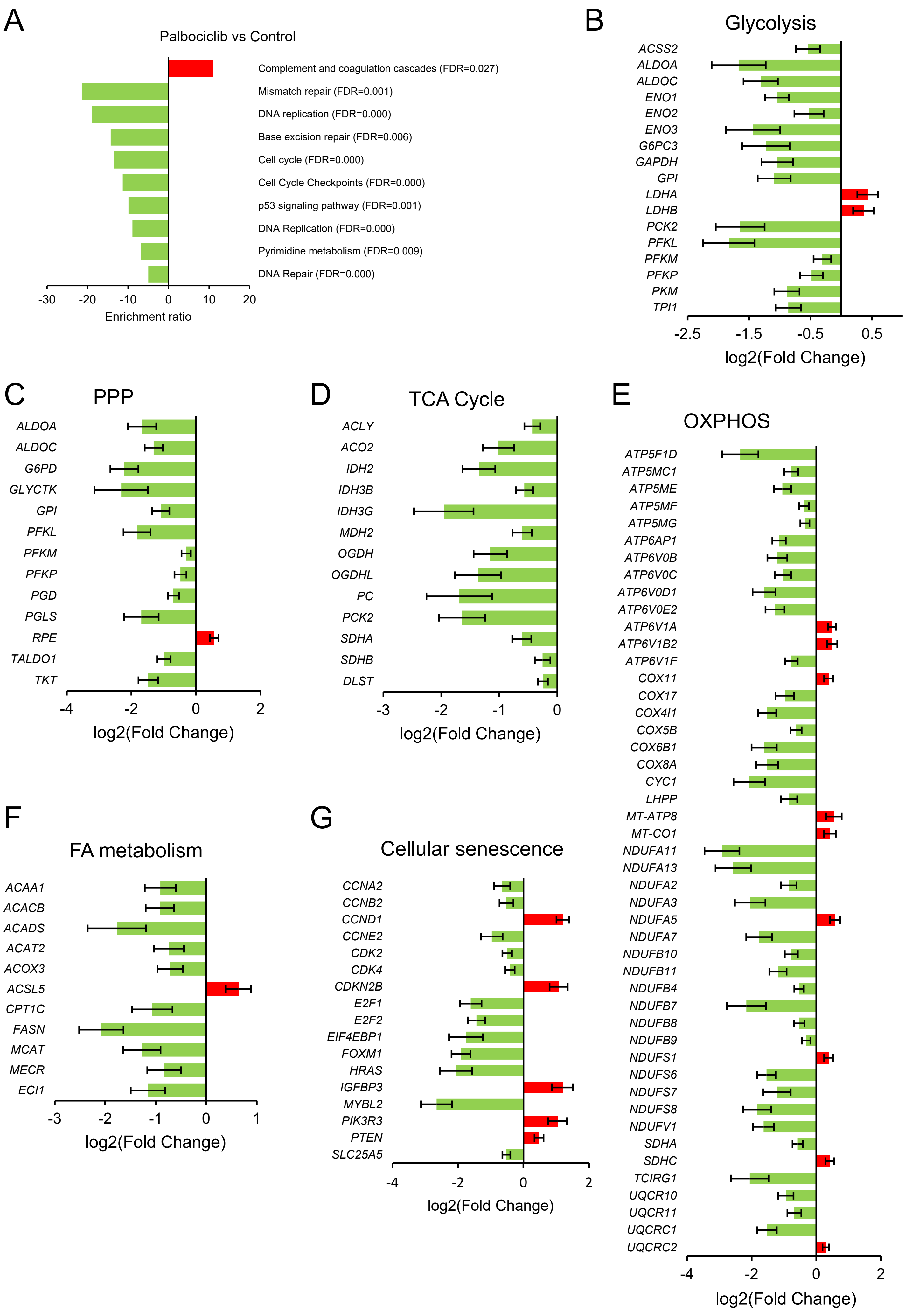

Supplementary Figure 6

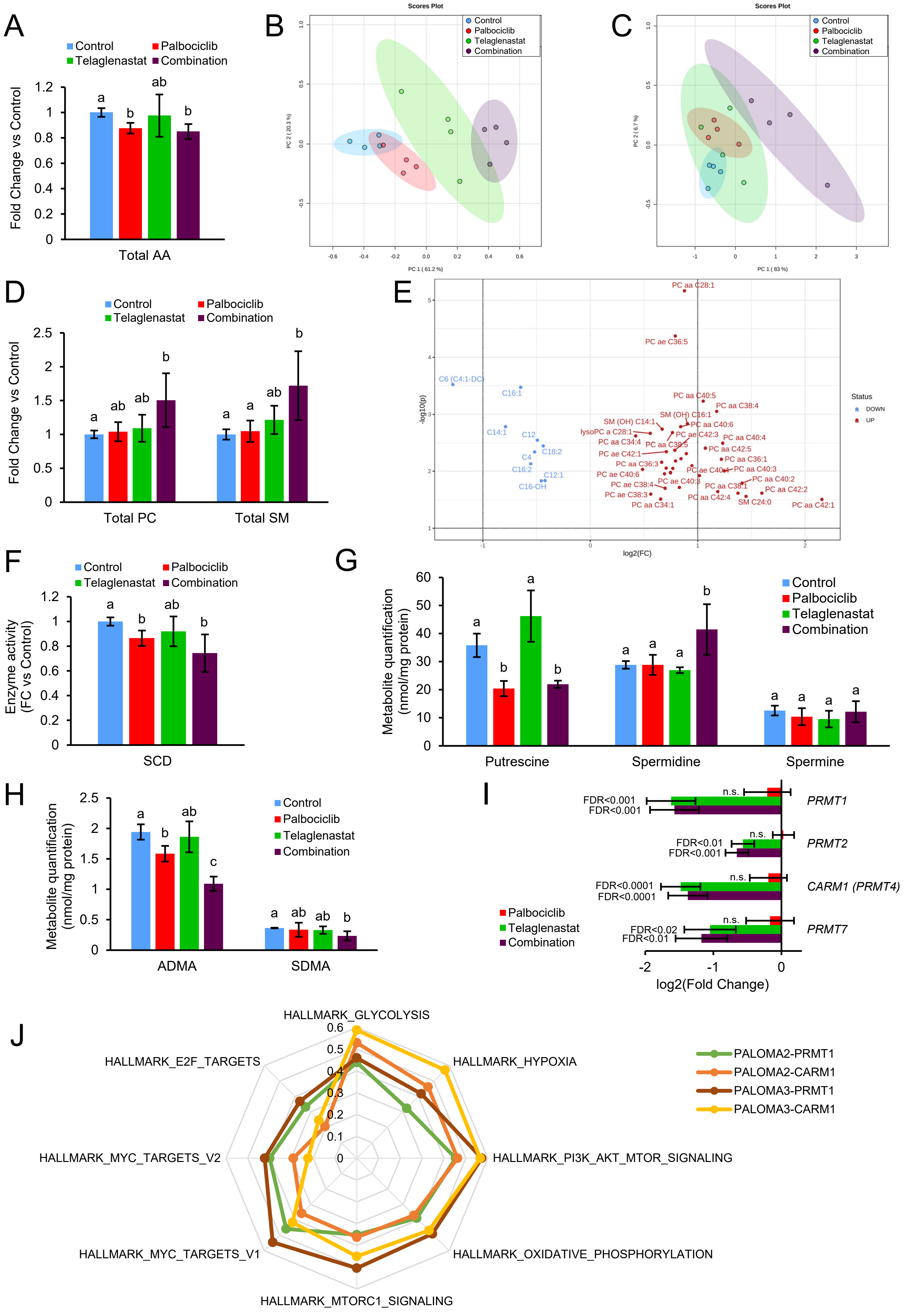

Supplementary Figure 6

K

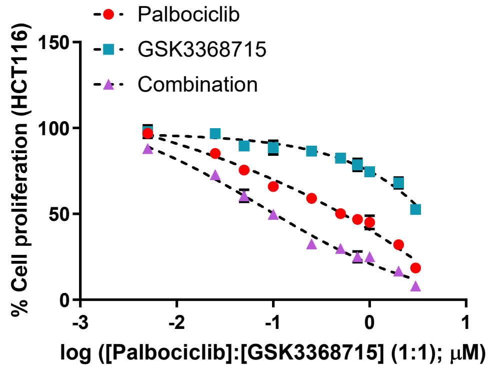

[Palbociclib] μM	[GSK3368715] μM	Viability	SD	CI Value
0.005	0.005	88.1	2.0	***0.244***
0.025	0.025	72.8	1.6	***0.256***
0.05	0.05	60.6	3.6	***0.219***
0.1	0.1	49.7	1.9	***0.223***
0.25	0.25	32.6	1.0	***0.188***
0.5	0.5	29.8	0.7	***0.308***
0.75	0.75	25.0	3.2	***0.320***
1	1	25.1	1.1	***0.430***
2	2	16.7	1.2	***0.395***
3	3	8.1	0.8	***0.169***

L

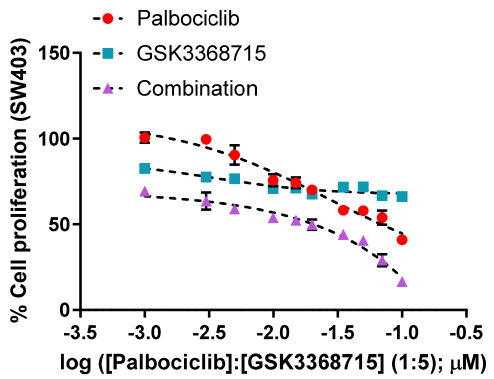

[Palbociclib] μM	[GSK3368715] μM	Viability	SD	CI Value
0.001	0.005	69.3	2.8	***0.092***
0.003	0.015	63.6	5.0	***0.139***
0.005	0.025	58.9	1.9	***0.148***
0.01	0.05	53.8	1.3	***0.193***
0.015	0.075	52.3	2.3	***0.257***
0.02	0.1	49.8	3.0	***0.282***
0.035	0.175	44.0	2.4	***0.315***
0.05	0.25	40.6	2.7	***0.344***
0.07	0.35	29.0	3.5	***0.182***
0.1	0.5	16.5	2.2	***0.066***

M

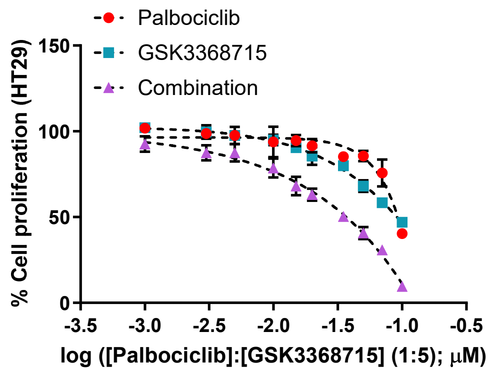

[Palbociclib] μM	[GSK3368715] μM	Viability	SD	CI Value
0.001	0.005	92.6	4.6	***0.124***
0.003	0.015	87.4	4.4	***0.228***
0.005	0.025	87.4	5.0	***0.382***
0.01	0.05	78.5	5.6	***0.455***
0.015	0.075	68.1	5.4	***0.446***
0.02	0.1	63.0	3.5	***0.499***
0.035	0.175	50.3	2.4	***0.586***
0.05	0.25	40.7	3.5	***0.627***
0.07	0.35	30.8	2.7	***0.637***
0.1	0.5	9.5	0.6	***0.321***

N

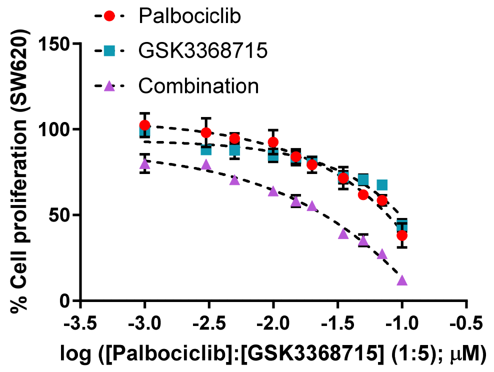

[Palbociclib] μM	[GSK3368715] μM	Viability	SD	CI Value
0.001	0.005	80.0	5.4	***0.110***
0.003	0.015	79.7	2.8	***0.321***
0.005	0.025	70.6	2.2	***0.273***
0.01	0.05	64.1	2.4	***0.370***
0.015	0.075	58.2	3.4	***0.408***
0.02	0.1	55.4	1.7	***0.473***
0.035	0.175	39.2	1.1	***0.387***
0.05	0.25	35.3	3.4	***0.460***
0.07	0.35	27.4	1.2	***0.434***
0.1	0.5	12.0	1.6	***0.223***

O

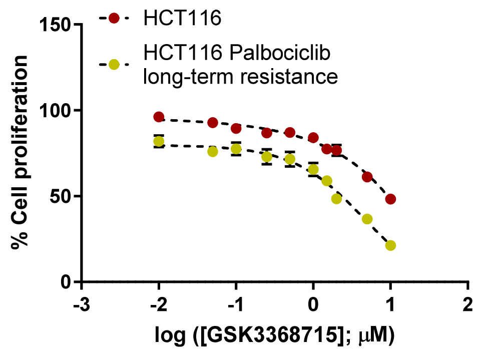

P

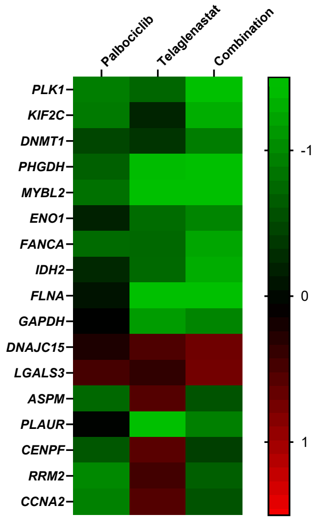

Supplementary Figure 7

A

Characterization of the metabolic adaptation to drug-induced stress

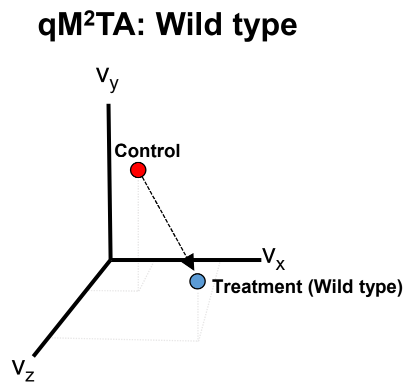

B

Identification of potential targets

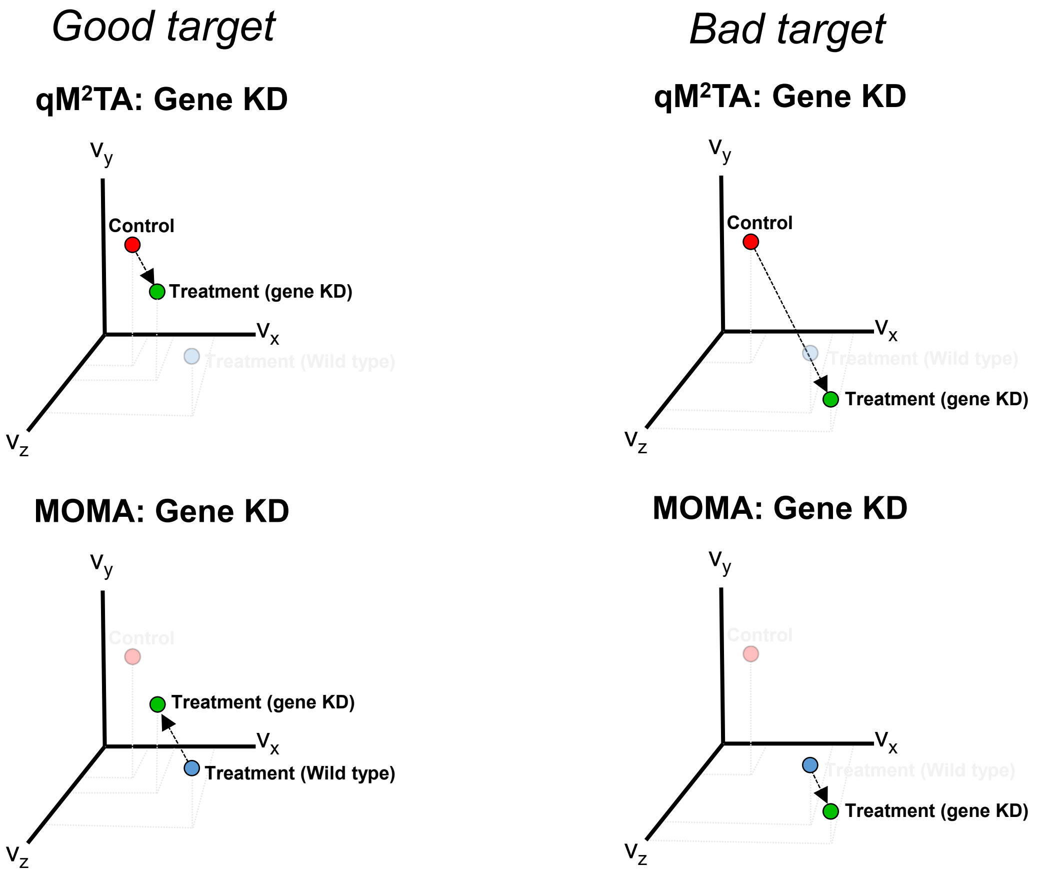

Supplementary Figure 8

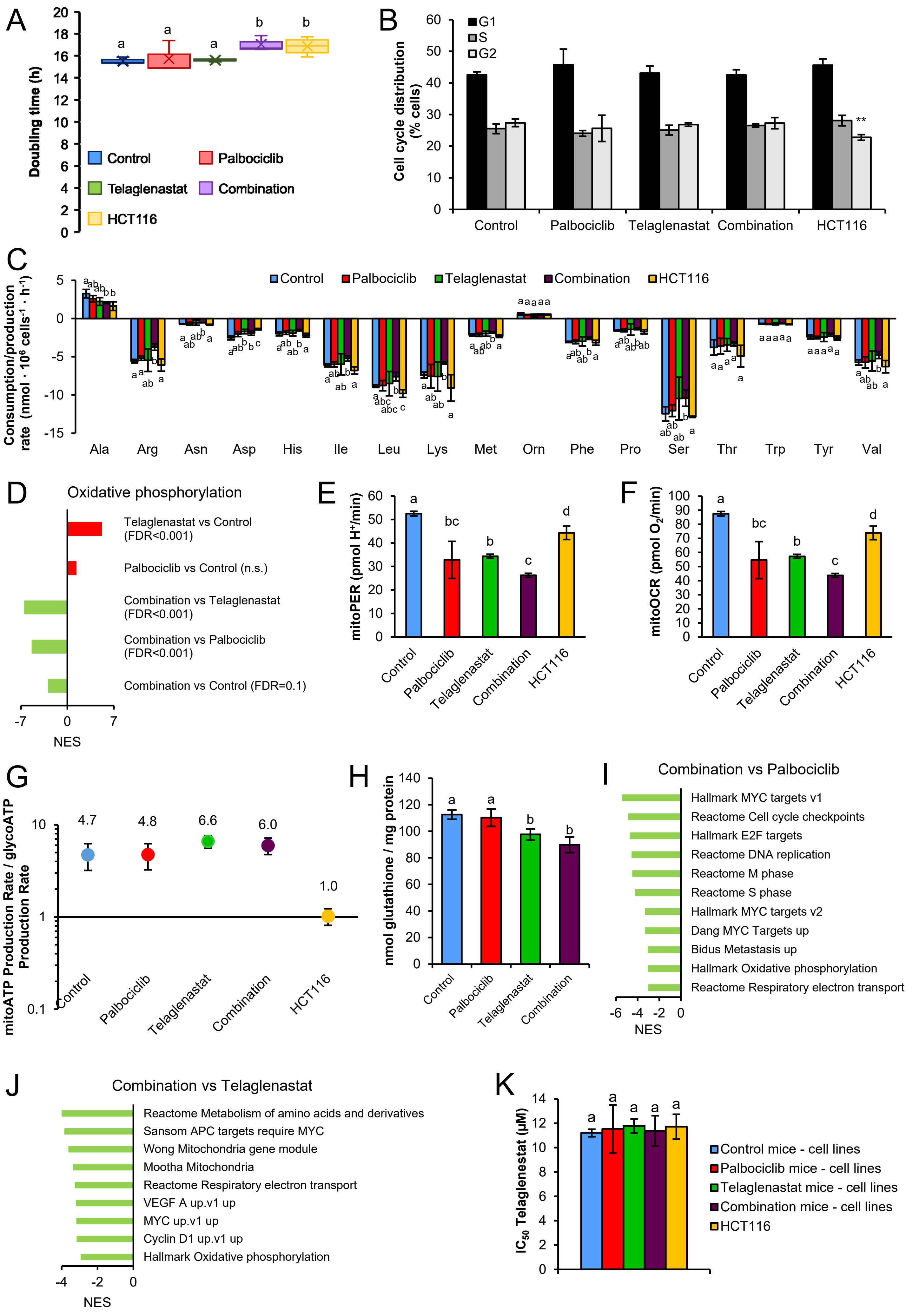

Supplement: Supplementary file 1 — Supplementary Figures [file 41388_2025_3495_MOESM1_ESM.pdf]
