# Supplementary material for: Glutaminase as a metabolic target of choice to counter acquired resistance to Palbociclib by colorectal cancer cells
Source: Oncogene. 2025 Jul 22;44(36):3386–406. doi: 10.1038/s41388-025-03495-w (PMC12399431; doi:10.1038/s41388-025-03495-w)
Supplement: Supplementary file 2 — Supplementary Figure and Table Legends [file 41388_2025_3495_MOESM2_ESM.docx]

**Supplementary Figure 1 (related to Figure 1).**

A. Left, the chemical structure of Palbociclib (PD0332991). Right, the chemical structure of Telaglenastat (CB-839).

B. Synergistic antiproliferative effect of Palbociclib and Telaglenastat combined treatment. HCT116 cells were treated for 96 hours at the indicated concentrations (µM) of inhibitors. The combination index (CI) results obtained with CompuSyn software (ComboSyn, Inc., Paramus, NJ, USA) revealed a strong synergy (CI<0.3) in the antiproliferative effects of Palbociclib and Telaglenastat at each dose combination tested.

C. Representative IncuCyte live-cell images in phase-contrast of HCT116 cells treated with Palbociclib (2 µM), Telaglenastat (5 µM), or their combination at 24, 48, 72, and 96 hours. Cell confluence was monitored every 3 hours. Scale bar = 400 μm.

D. Spheroid formation assay. Left, scan (top) and phase-contrast microscope (60x, bottom) images of HCT116 spheroids grown on anchorage-independent conditions and treated for 10 days with Palbociclib (2 µM), the glutaminase inhibitors BPTES (10 µM) and Telaglenastat (5 µM), or their combination. Right, quantification of total spheroid volume. Spheroids were scored by image acquisition, followed by spheroid area and volume quantification with ImageJ. Results are represented as the percentage of total spheroid volume relative to untreated cells (mean ± SD of n=4).

E. Colony-formation assay. Left, images of HCT116 cells cultured in the absence or presence of 2 µM Palbociclib, 5 µM Telaglenastat, or their combination for 10 days. Right, quantification of the total number of colonies scored by image acquisition with ImageJ.

F-H. Cell proliferation assay. SW403 (F), HT29 (G), and SW620 (H) cells were treated with constant-ratio increasing concentrations of Palbociclib, Telaglenastat, or their combination for 96 hours, and cell quantification was assessed by Hoechst 33342 staining. Results are shown as the percentage of proliferation relative to untreated cells (mean ± SD for n=6). Top, concentration-response curves generated by data fitting with a four-parameter equation, and associated combination index (CI) values table. Bottom, dose-response cell proliferation matrix, and dose-response synergy matrix. The CI values obtained with CompuSyn software depicted a synergistic antiproliferative effect (CI<0.9) at each dose combination tested.

I. Schematic outline of the experimental procedure for the determination of the extracellular metabolic fluxes for HCT116 cells at 96 and 192 hours of treatment with Palbociclib, Telaglenastat, or their combination.

J. Quantification of intracellular metabolites related to the mitochondrial metabolism after 96 hour-treatment with 2 µM Palbociclib assessed by HPLC-MS. Results are shown as the percentage of total content relative to control cells (mean ± SD for n=3). Significance was determined by two-tailed independent sample Student’s t-tests. Statistically significant differences between treated and control cells are indicated as *P* < 0.05 (*), *P* < 0.01 (**), and *P* < 0.001 (***).

K. Box and whiskers plot of doubling time of HCT116, MCF-7, SK-BR-3, and MDA-MB-231 cells treated with Palbociclib, Telaglenastat, or their combination in the presence or absence of dimethyl α-KG (DM-αKG). Shown values are the mean ± SD of three independent experiments with four replicates each. Statistically significant differences between conditions are indicated with different letters.

L. Cell proliferation curves for MCF-7, SK-BR-3, and MDA-MB-231 cells treated with Palbociclib, Telaglenastat, or their combination, in the presence or absence of dimethyl α-KG (DM-αKG) for 96 hours. Confluence was monitored in the Olympus CM30 Incubation Monitoring System (Evident Corporation). Results are shown as mean ± SD with n=6. Statistically significant differences between conditions are indicated with different letters.

M, N. Cell proliferation assay with Palbociclib (M) or the glutaminase inhibitors Telaglenastat, BPTES, DON, Compound 968, Glutaminase-IN-1 (GLS-IN-1) (N) in parental HCT116 cells and HCT116 cells with long-term Palbociclib resistance, which were established by routinely culturing HCT116 cells in the presence of Palbociclib for four months. Cells were treated with constant-ratio increasing concentrations of Palbociclib or each glutaminase inhibitor for 96 hours and cell quantification was assessed by Hoechst 33342 staining. Results are shown as the percentage of proliferation relative to untreated cells (mean ± SD for n=6).

**Supplementary Figure 2 (related to Figure 2).**

A. Schematic outline of the experimental procedure for testing the combined therapy *in vivo*: NOD-SCID mice were injected subcutaneously with HCT116 cells (1 × 10^6^ cells/mouse) (Day 0). After seven days of tumor expansion, the mice were randomized into four groups and treated daily for 23 days with vehicle (Control), Palbociclib (PD0332991), Telaglenastat (CB-839), or their combination (PD0332991+CB-839) (n=12 per group). Tumor volume was measured with a Vernier caliper at the indicated days (orange circles). On Day 31, mice were euthanized, and tumors were extracted.

B. Mean mouse weights for each treatment group were measured every 2-3 days (n=12 per group). Data are presented as mean ± SD. Yellow shadow delimits the growth curve range of male NOD.CB17-Prkdcscid/scid/Rj mice (data from Janvier Labs).

C. Final body weight of individual mice at the end of the treatments (n=12 per group). Data are represented in a box and whiskers plot, with the whiskers representing the minimum and maximum values, all data points shown, and the median indicated.

D. Percentage change in mouse weights from the start of the experiment normalized to the weight of Day 0. Data are shown as mean ± SD.

E. Percentage change in individual mice weights at the end of the treatments (n=12 per group). Data are represented as mean ± SD.

F. Representative bioluminescence imaging of mice with luciferase-expressing HCT116 subcutaneous colorectal cancer xenografts after D-luciferin injection. Tumor progression and treatment effects were monitored by 2D bioluminescence imaging (BLI) of average radiance (photons (p)/s/cm^2^/steradian (sr)).

G. Representative histopathology images of Hematoxylin and Eosin (H & E) stained human colon carcinoma xenografts generated from HCT116 cells; magnification x500, scale bar = 20 μm. Circles indicate mitotic figures.

H-J. Pearson linear correlation between %CD31 and %KI67 positive cells (G), mitotic count and %KI67 positive cells (H), and mitotic count and %CD31 positive cells (I).

Data information: Significance was determined by ANOVA and Tukey’s multiple comparisons test with α=0.05. Statistically significant differences between conditions are indicated with different letters.

**Supplementary Figure 3 (related to Figure 3).**

A. Volcano plots illustrating the differential gene expression between Palbociclib, Telaglenastat, or the combined treatment and control tumors. The x-axis corresponds to the magnitude of change (log2 of the fold change), and the y-axis is the statistical significance (-log10 of the false discovery rate adjusted P-value (FDR)). Genes with FDR < 0.05 were considered statistically significant.

B. Volcano plots depicting the metabolic genes differentially expressed between Palbociclib, Telaglenastat, or the combined treatment and control tumors. Metabolic genes are defined as genes that are associated with reactions or transport processes in the Recon3D genome-scale reconstruction of human metabolism.

C. Top-ranked biological processes affected by Palbociclib, Telaglenastat, or the combined treatments obtained by the transcriptome profiling of HCT116 tumors. The number of differentially expressed (FDR < 0.05) genes assigned to each process is reported next to the bars.

D. Volcano plots portraying the metabolic genes differentially expressed between Palbociclib or Telaglenastat and the combined treatment.

**Supplementary Figure 4 (related to Figure 3). Volcano plots of over-representation analysis (ORA) results.** Volcano plots representing the -log10 of FDR against enrichment ratio or NES for all the categories in the search database. Significant categories are near the upper corners. The size and color intensity of the dots are proportional to the number of overlapping genes between the gene set of the category and the differentially expressed genes of each treatment.

A. Downregulated gene sets in tumors treated with Palbociclib.

B. Upregulated gene sets in tumors treated with Palbociclib.

C. Upregulated gene sets in tumors treated with Telaglenastat.

D. Downregulated gene sets in tumors treated with Telaglenastat.

E. Downregulated gene sets in tumors treated with the combination of Palbociclib and Telaglenastat.

F. Upregulated gene sets in tumors treated with the combination of Palbociclib and Telaglenastat.

**Supplementary Figure 5 (related to Figure 3). Transcriptomic analysis of Palbociclib, Telaglenastat, and their combination treatments *in vivo*.**

A. Over-representation analysis (ORA) of downregulated (green) or upregulated (red) gene sets in tumors treated with Palbociclib for 23 days (FDR < 0.05).

B-G. Genes related to glycolysis (B), pentose phosphate pathway (PPP) (C), tricarboxylic acid (TCA) cycle (D), oxidative phosphorylation (OXPHOS) (E), fatty acid (FA) metabolism (F), and cellular senescence (G) that are differentially expressed (*p* adjusted < 0.05) in HCT116 tumors treated with the combination of Palbociclib and Telaglenastat compared to control tumors.

**Supplementary Figure 6 (related to Figure 4). Tumor metabolic profiling.** The metabolic quantification of tumors treated with vehicle, Palbociclib, Telaglenastat, or the combination of Palbociclib and Telaglenastat was determined by tandem mass spectrometry coupled to liquid chromatography (LC/MS/MS) or flow injection analysis (FIA/MS/MS).

A. Quantification of total amino acids (AA) in tumors treated with Palbociclib, Telaglenastat, or the combination of Palbociclib and Telaglenastat relative to control tumors.

B. Principal component analysis of the amino acid metabolic profiling of the tumors treated with vehicle, Palbociclib, Telaglenastat, or the combination of Palbociclib and Telaglenastat.

C. Principal component analysis of the lipidomic profiling of the tumors treated with vehicle, Palbociclib, Telaglenastat, or the combination of Palbociclib and Telaglenastat.

D. Quantification of total phosphatidylcholines (PC) and sphingomyelins (SM) in tumors treated with Palbociclib, Telaglenastat, or the combination of Palbociclib and Telaglenastat relative to control tumors.

E. Volcano plot (-log10(P-value) vs log2(FC)) of the significant (P-value < 0.05) changes in lipid metabolites between tumors treated with the combination of Palbociclib and Telaglenastat and control tumors. For phosphatidylcholines (PC), the total number of carbon atoms and double bonds of the diacyl (aa) or acyl–alkyl (ae) groups is represented by Cx:y, where x indicates the number of carbons and y the number of double bonds. The same notation is used for describing the length and the number of double bonds in the acyl chain of acylcarnitines (C), lysophosphatidylcholines (lysoPC), sphingomyelins (SM) and hydroxylated sphingomyelins (SM (OH)).

F. Estimation of stearoyl CoA-desaturase (SCD) enzyme activity relative to the control condition.

G. Quantification of putrescine, spermidine, and spermine in tumors treated with vehicle, Palbociclib, Telaglenastat, or the combination of Palbociclib and Telaglenastat.

H. Quantification asymmetric dimethylarginine (ADMA) and symmetric dimethylarginine (SDMA) in tumors treated with vehicle, Palbociclib, Telaglenastat, or the combination of Palbociclib and Telaglenastat.

I. Protein arginine methyltransferases (PRMTs) genes differentially expressed (*p* adjusted < 0.05) in tumors treated with Palbociclib, Telaglenastat, or the combination of Palbociclib and Telaglenastat compared to control tumors.

J. Pearson’s correlation between the enrichment of hallmark gene sets associated with Palbociclib resistance and the gene expression of *PRMT1* and *CARM1* in PALOMA-2/3 clinical trials.

K-N. Cell proliferation assay for the combination of Palbociclib and the PRMT type I inhibitor GSK3368715. HCT116 (K), SW403 (L), HT29 (M), and SW620 (N) cells were treated with constant-ratio increasing concentrations of Palbociclib, GSK3368715, or their combination for 96 hours, and cell quantification was assessed by Hoechst 33342 staining. Results are shown as the percentage of proliferation relative to untreated cells (mean ± SD for n=6). Left, concentration-response curves generated by data fitting with a four-parameter equation. Right, associated combination index (CI) values tables. The CI values obtained with CompuSyn software depicted a synergistic antiproliferative effect (CI<0.7) at each dose combination tested.

O. Cell proliferation assay with the PRMT type I inhibitor GSK3368715 in parental HCT116 cells and HCT116 cells with long-term Palbociclib resistance, which were established by routinely culturing HCT116 cells in the presence of Palbociclib for four months. Cells were treated with constant-ratio increasing concentrations of GSK3368715 for 96 hours and cell quantification was assessed by Hoechst 33342 staining. Results are shown as the percentage of proliferation relative to untreated cells (mean ± SD for n=6).

P. Heatmap of the expression of genes associated with Palbociclib resistance, as identified in the PEARL clinical trial, in tumors treated with Palbociclib, Telaglenastat, or the combination of Palbociclib and Telaglenastat. Only the genes that are differentially expressed (p adjusted < 0.05) in HCT116 tumors treated with the combination of Palbociclib and Telaglenastat compared to control tumors are shown.

Data information: Significance was determined by one-way ANOVA and Tukey’s multiple comparisons test with α=0.05. Statistically significant differences between conditions are indicated with different letters (except where stated otherwise). Shown values are mean ± SD for n=4.

**Supplementary Figure 7 (related to Figure 5). Quadratic Metabolic transformation Algorithm (qM^2^TA).**

A. qM^2^TA applied to the characterization of the metabolic adaptation to drug-induced stress. qM^2^TA is used to simulate the flux changes associated with adaptation to drug-induced stress starting from the control. A hypothetical transition is shown in a 3D space for the flux through the reactions (v_x_, v_y_, v_z_).

B. Identification of potential targets. Targets are evaluated by simulating the capacity of gene knockdowns (KD) to inhibit the metabolic transformation (qM^2^TA: Gene KD) and reverting the drug-adapted metabolic state (MOMA: Gene KD). A good target would be one that partially impedes the metabolic transformation from control to drug-induced stress while also allowing to partially revert the drug-induced stress metabolic state to the control state.

**Supplementary Figure 8 (related to Figure 6). Characterization of cell lines derived from HCT116 xenografts.** Twelve cell lines were obtained from HCT116 tumors from mice that had been treated daily with vehicle, Palbociclib, Telaglenastat, or the combination of Palbociclib and Telaglenastat for 23 days (three cell lines per condition). All cell lines were grown in the absence of chemotherapeutics.

A. Cell doubling time of cell lines derived from tumors that had been treated with vehicle, Palbociclib, Telaglenastat, or the combination of Palbociclib and Telaglenastat, and parental HCT116 cells.

B. Cell cycle distribution in cell lines derived from tumors that had been treated with vehicle, Palbociclib, Telaglenastat, or the combination of Palbociclib and Telaglenastat, and parental HCT116 cells determined by flow cytometry. Statistically significant differences between treated and control cells are indicated as P < 0.05 (*), P < 0.01 (**), and P < 0.001 (***).

C. Amino acid consumption and production rates measured by tandem mass spectrometry coupled to liquid chromatography (LC/MS/MS) after 24 hours of incubation with fresh media and normalized to cell number.

D. Normalized enrichment scores for the oxidative phosphorylation gene set between the cell lines derived from tumors.

E. Mitochondria-derived acidification (mitoPER) assessed with a Seahorse analyzer. Data are normalized to cell number and shown as the mean of three independent cell lines for each condition ± SD with n=5.

F. Mitochondrial oxygen consumption rate (mitoOCR) measured with a Seahorse analyzer. Data are normalized to cell number and shown as the mean of three independent cell lines for each condition ± SD with n=5.

G. Representation of the metabolic index (or ATP rate index) as the ratio of the mitochondrial ATP (mitoATP) production rate to the glycolytic ATP (glycoATP) production rate as a quantitative metric of the cellular metabolic phenotype.

H. Total glutathione quantification normalized to protein content for the cell lines derived from tumors that had been treated with vehicle, Palbociclib, Telaglenastat, or the combination of Palbociclib and Telaglenastat.

I-J. Gene set enrichment analysis (GSEA) of cells obtained from tumors subjected to the combined therapy compared with cells derived from tumors treated with Palbociclib (I) or Telaglenastat (J) alone.

K. IC_50_ values for Telaglenastat in cell lines derived from residual tumors from mice that had been treated with vehicle, Palbociclib, Telaglenastat, and the combination of Palbociclib and Telaglenastat *in vivo*. Bars represent the mean of the IC_50_ values of three independent cell lines for each condition ± SD with n=6.

Data information: Ala, alanine; Arg, arginine; Asn, asparagine; Asp, aspartate; Cit, citrulline; Gln, glutamine; Glu, glutamate; Gly, glycine; His, histidine; Ile, isoleucine; Leu, leucine; Lys, lysine; Met, methionine; Orn, ornithine; Phe, phenylalanine; Pro, proline; Ser, serine; Thr, threonine; Trp, tryptophan; Tyr, tyrosine; Val, valine. Shown values are the mean of three independent cell lines for each condition ± SD for n=3 (except otherwise indicated). Significance was determined by one-way ANOVA and Tukey’s multiple comparisons test with α=0.05. Statistically significant differences between conditions are represented with different letters.

**Supplementary Table 1. Summary statistics of the RNA-sequencing differential gene expression analysis performed *in vivo* and *ex vivo*.** The analysis was performed with the DESeq2 package for R and P-values were adjusted for multiple testing using the FDR method. Metabolic genes, defined as genes that are associated with reactions or transport processes in the Recon3D genome-scale reconstruction of human metabolism, are indicated.

**Supplementary Table 2. Correlation between the expression of *PRMT1* and *CARM1* genes and the gene sets enrichment in PI3K/AKT/mTOR signaling, OXPHOS, MYC signaling, mTOR signaling, hypoxia, glycolysis, and E2F signaling pathways in the PALOMA 2/3 clinical trials.** Gene enrichment was computed with the ssGSEA2 package for R, and Pearson correlation was evaluated with the cor.test function. The resulting P-values were adjusted for all tested pairs using the FDR method.

**Supplementary Table 3. Correlation between the expression of *PRMT1* and *CARM1* genes and genes associated with Palbociclib resistance or sensitivity in the PALOMA 2/3 clinical trials.** Pearson Correlation was evaluated with the cor.test function from R. The resulting P-values were adjusted for all tested pairs using the FDR method. Palbociclib resistance genes were identified in PALOMA-2/3 trials comparative biomarker analyses (57, 58).

**Supplementary Table 4. Reaction and pathway flux values computed with the quadratic metabolic transformation algorithm.** Simulated flux values in xenograft tumors for individual reactions and pathways and their variation in response to Palbociclib, Telaglenastat, or their combination are provided. Flux values were obtained by integrating transcriptomics and metabolomics with the quadratic metabolic transformation algorithm and have arbitrary units.

**Supplementary Table 5. Gene target scores computed with the quadratic metabolic transformation algorithm.** Gene target scores represent the predicted capacity of a gene knockdown to revert the metabolic adaptation to Palbociclib or Telaglenastat. Any gene with a positive score is a putative target. Genes that are significantly downregulated (adjusted P-value<0.05; Supplementary Table 1) at the gene expression level by the reciprocal treatment are also indicated.

**Supplementary Table 6. The synergistic antiproliferative effect of Palbociclib** **and Telaglenastat** **combined treatment is conserved *ex vivo*.** Cells obtained from tumors from mice that have been administered with Palbociclib and Telaglenastat combination were treated for 96 hours at the indicated concentrations (µM) of Palbociclib and Telaglenastat in a constant ratio (1:10). The combination index (CI) results obtained with CompuSyn software revealed a synergy (CI<1) in the antiproliferative effects of Palbociclib and Telaglenastat at each dose combination tested.
