# Supplementary material for: Glutaminase as a metabolic target of choice to counter acquired resistance to Palbociclib by colorectal cancer cells
Source: Oncogene. 2025 Jul 22;44(36):3386–406. doi: 10.1038/s41388-025-03495-w (PMC12399431; doi:10.1038/s41388-025-03495-w)
Supplement: Supplementary file 8 — Supplementary Table 6 [file 41388_2025_3495_MOESM8_ESM.pdf]

Supplementary Table 6. The synergistic antiproliferative effect of Palbociclib and Telaglenastat combined treatment is conserved *ex vivo.* Cells obtained from tumors from mice that have been administered with Palbociclib and Telaglenastat combination were treated for 96 h at the indicated concentrations (μM) of Palbociclib and Telaglenastat in a constant ratio (1:10). The combination index (CI) results obtained with CompuSyn software revealed a synergy (CI<1) in the antiproliferative effects of Palbociclib and Telaglenastat at each dose combination tested.

Combination Index (CI) equation of Chou and Talalay and CompuSyn software

CI value	Agonistic effect
<0.10	Very strong synergism
0.10–0.30	Strong synergism
0.30–0.70	Synergism
0.70–0.90	Moderate to slight synergism
0.90–1.10	Nearly additive
1.10–1.45	Slight to moderate antagonism
1.45–3.30	Antagonism
>3.30	Strong to very strong antagonism

		Cell line 1 derived from tumor 1 treated with Combination			Cell line 2 derived from tumor 2 treated with Combination			Cell line 3 derived from tumor 3 treated with Combination		
[Palbociclib] μM	[Telaglenastat] μM	Viability	SD	CI Value	Viability	SD	CI Value	Viability	SD	CI Value
0.04	0.4	80.9	1.5	***0.450***	76.8	1.0	***0.615***	78.6	1.9	***0.662***
0.08	0.8	71.0	2.3	***0.398***	64.7	5.6	***0.386***	67.0	2.7	***0.400***
0.15	1.5	59.7	3.3	***0.367***	55.6	2.0	***0.379***	57.2	1.3	***0.350***
0.3	3	52.2	2.0	***0.486***	48.9	5.2	***0.514***	48.9	4.8	***0.404***
0.5	5	50.7	5.4	***0.749***	47.9	3.0	***0.813***	48.1	1.1	***0.641***
0.65	6.5	49.0	2.4	***0.891***	43.9	2.5	***0.869***	43.5	0.8	***0.632***
0.8	8	42.8	1.5	***0.797***	41.0	5.0	***0.940***	41.8	3.3	***0.704***
0.9	9	37.9	3.5	***0.698***	35.0	2.6	***0.834***	37.7	3.6	***0.627***
1.1	11	27.0	1.6	***0.476***	26.3	1.8	***0.764***	17.7	1.0	***0.238***
1.4	14	13.5	2.1	***0.245***	11.1	2.4	***0.616***	8.6	1.7	***0.140***
